# A computational psychiatry approach identifies how alpha-2A noradrenergic agonist Guanfacine affects feature-based reinforcement learning in the macaque

**DOI:** 10.1038/srep40606

**Published:** 2017-01-16

**Authors:** S. A. Hassani, M. Oemisch, M. Balcarras, S. Westendorff, S. Ardid, M. A. van der Meer, P. Tiesinga, T. Womelsdorf

**Affiliations:** 1 Department of Biology, Centre for Vision Research, York University, Toronto, Ontario M6J 1P3, Canada; 2 Department of Mathematics, Boston University, Boston, MA 02215, USA; 3 Department of Psychological and Brain Sciences, Dartmouth College, Hanover, NH 03755, USA; 4 Department of Neuroinformatics, Donders Centre for Neuroscience, Radboud University Nijmegen, Nijmegen, AJ 6525, The Netherlands

## Abstract

Noradrenaline is believed to support cognitive flexibility through the alpha 2A noradrenergic receptor (a2A-NAR) acting in prefrontal cortex. Enhanced flexibility has been inferred from improved working memory with the a2A-NA agonist Guanfacine. But it has been unclear whether Guanfacine improves specific attention and learning mechanisms beyond working memory, and whether the drug effects can be formalized computationally to allow single subject predictions. We tested and confirmed these suggestions in a case study with a healthy nonhuman primate performing a feature-based reversal learning task evaluating performance using Bayesian and Reinforcement learning models. In an initial dose-testing phase we found a Guanfacine dose that increased performance accuracy, decreased distractibility and improved learning. In a second experimental phase using only that dose we examined the faster feature-based reversal learning with Guanfacine with single-subject computational modeling. Parameter estimation suggested that improved learning is not accounted for by varying a single reinforcement learning mechanism, but by changing the set of parameter values to higher learning rates and stronger suppression of non-chosen over chosen feature information. These findings provide an important starting point for developing nonhuman primate models to discern the synaptic mechanisms of attention and learning functions within the context of a computational neuropsychiatry framework.

Attentional flexibility is compromised in many neuropsychiatric diseases and becomes manifest in perseverative behaviours, impulsivity, poor set-shifting abilities, or higher distractibility. These cognitive effects can be experimentally dissociated in reversal learning tasks, providing a rich test-bed to identify the neuromodulatory and synaptic mechanisms that support flexible attention during reversal learning. Previous studies have implicated, in particular, dopaminergic and noradrenergic signaling in prefrontal-striatal loops to support cognitive flexibility^1^,^2^,^3^. One prominent receptor subtype involved is the alpha 2A noradrenergic receptor (a2A-NAR) whose activation at optimal concentrations enhances working memory representations in prefrontal cortex (PFC) by increasing neuronal firing of memorized target locations^4^,^5^. Such an enhanced delay firing during a2A-NAR activation could be the correlate for enhanced flexibility during goal-directed behaviour.

However, how improved working memory representations relate to otherwise dissociable measures of behavioural flexibility, such as reduced impulsivity, reduced distractibility from irrelevant salient events, enhanced attentiveness/vigilance, improved sensitivity to salient events, or heightened sensitivity to behavioural outcomes to adjust behaviour in the light of errors has remained elusive. All these cognitive subfunctions render behaviour flexible and have also been linked to catecholaminergic action in the PFC. For example, noradrenergic activation has been implicated to balance the relative weighting of explorative tendencies over exploitative tendencies during periods of uncertainty^6^,^7^, and to enhance the focusing on relevant sensory information^8^,^9^. Such influences of noradrenergic action could act in addition to changes in working memory and could have complex behavioural effects that have not only benefits, but also costs to behavioural performance. For example, favoring exploratory choices can enhance performance and reduces perseverative tendencies in uncertain situations, but it can also introduce noise and thereby reduce performance when the environment does not change akin to enhanced distractibility^10^.

To understand the specific cognitive consequences of noradrenergic action on goal-directed behaviour it seems therefore pivotal to study selective receptor systems in a variety of tasks. Guanfacine is a selective a2A-NAR agonist with low affinity for the receptor subtypes alpha 2B and 2C^11^. For the selective a2A-NAR system, studies in rodents and nonhuman primates suggest that certain doses improve working memory (e.g. refs 12, 13, 14), as well as decrease impulsivity, reduce distractibility and possibly facilitate faster, more consistent learning^15^,^16^,^17^. At the molecular level, Guanfacine preferentially binds to post-synaptic alpha 2A receptors^18^. Pyramidal cells in prefrontal regions richly express post-synaptic alpha 2A receptors^19^, and stimulation of these receptors is thought to inhibit cyclic adenosine monophosphate (cAMP) production, which leads to closing of nearby HCN channels, which in turn leads to increased excitability in prefrontal pyramidal cells and increased connectivity within prefrontal microcircuits^5^,^20^. Guanfacine is suggested to exert its positive effects on cognitive functions via these actions on post-synaptic a2A-NAR receptors in the dorsolateral PFC^5^. Guanfacine is also suggested to suppress glutamatergic synaptic transmission and thereby neural excitability at deeper layers (V/VI) in PFC, potentially governed by similar intra-cellular mechanisms as those controlling HCN channels^21^,^22^. It has been proposed that at low concentrations, Guanfacine’s actions on HCN channels may predominate, while only at high concentrations glutamate transmission is affected, potentially explaining an inverted-U type function of Guanfacine^5^,^22^. Guanfacine also binds to pre-synaptic alpha 2A receptors on locus coeruleus terminals that act as inhibitory auto-receptors, thereby decreasing NE release^23^, which may again suggest that high doses of Guanfacine could impair cognitive functions.

The evidence of positive effects seen with Guanfacine in rodents and non-human primates is not easily reconciled with results from healthy human subjects, where the influence of single doses of Guanfacine on behavioural flexibility is inconclusive. Some studies report improved planning performance, improved working memory, and improved paired-associates learning^24^,^25^, while other studies did not see changes with Guanfacine on a broad range of executive function tests including spatial working memory, problem solving, intra-/extra- dimensional attentional shift and behavioural inhibition tasks^26^. This mixture of results in healthy humans following administration of a single dose contrasts to those from ADHD diagnosed subject groups in which Guanfacine has been found at the group level to improve interference control (Stroop task), and to enhance sustained attention in the continuous performance task (e.g. refs 27,28). These task improvements in clinical populations reflect enhanced attentiveness (i.e. detecting more target stimuli, showing less omission errors) and reduced impulsiveness (i.e. higher capability to correctly withhold responding to non-target stimuli, less commission errors)^27^.

Here, we apply a computational psychiatry approach to understanding a2A-NA drug action on higher cognitive functions, examining how a formal framework can add clarity to the complex empirical state of a2A-NA effects. Computational psychiatry includes as one branch quantitative Bayesian and Reinforcement learning (RL) modeling of drug actions on higher cognitive functioning^28^. Testing formal Bayes and RL models of drug action promises critical benefits over non-formal approaches. Firstly, they come with statistical tools of model selection and validation, thereby making it possible to quantitatively test different theoretical constructs. Secondly, common model parameters provide a common language that facilitates comparisons between different studies, task paradigms, and subject groups. Thirdly, quantitative model selection enables single subject predictions of behavioral drug effects^29^. We utilize these benefits of a computational framework to identify which model and model parameters best account for alpha 2A influences on the performance of a healthy macaque monkey in a feature-based reversal learning task.

## Results

We reviewed the literature in order to identify non-human primate studies where systemically delivered Guanfacine improved cognitive performance (Table 1). The literature showed no consensus in the concentration of Guanfacine that produced observable improvement in a number of various behavioral paradigms. Furthermore, between the tasks used to test the efficacy of Guanfacine, there seemed to be considerable variation amongst the cognitive demands required for the performance of each task. This leads us to conclude that there is a lack of clarity revolving the specific cognitive change brought about by Guanfacine that leads to behavioral improvement. Across the 11 studies we found we broke down the (n = 14) behavioral paradigms used to evaluate Guanfacine into six temporally sequenced processing demands: stimulus encoding, working memory, attentional or interference control, choice/stimulus response mapping, learning requirement and formalized decision variable. Not all processing demands were present in every behavioral paradigm, of the 14 tasks in the 11 studies, 5 tasks had a demand on working memory, 11 tasks explicitly required attention or interference control, 8 defined a learning requirement and only 1 study quantified the influence of a formal decision variable using a computational model (see Table 1). Notably, of the 9 tasks that did not contain an explicit working memory component, 7 reported improvements using Guanfacine with at least one concentration tested. This survey result suggests that there are likely multiple routes through which Guanfacine affects goal directed behavior in addition to working memory.

We also noted the concentration used by each study, whether the study involved aged primates, and noted if an effect was or was not found using that concentration (Table 1). The concentrations with which task-related improvement was observed ranged from 0.00001 mg/kg to 0.2 mg/kg. The concentration range was broad for studies that used both aged and non-aged primates. Studies suggest that higher concentrations of Guanfacine shift its locus of action from post-synaptic to pre-synaptic a2A-NAR^30^. Pre-synaptic a2A-NAR’s are present in the locus coeruleus (LC) and act as inhibitory auto-receptors reducing NE release throughout the cortex^31^. This suggests that depending on the concentration of Guanfacine used, different adjustments to behavior may result from pre-synaptic a2A-NAR driven shifts of NE concentrations^5^, which may help explain the variability seen in Table 1. From a general perspective this survey illustrates that Guanfacine can improve performance for healthy monkeys at different age groups, for tasks requiring multiple different processing components, and for concentration ranges benefitting behavior that are highly variable and presumably subject specific. We believe that this lends power to single subject studies in which careful analysis of the cognitive change from Guanfacine borne improvement can help inform us of its mechanism of action.

**Figure 1:**
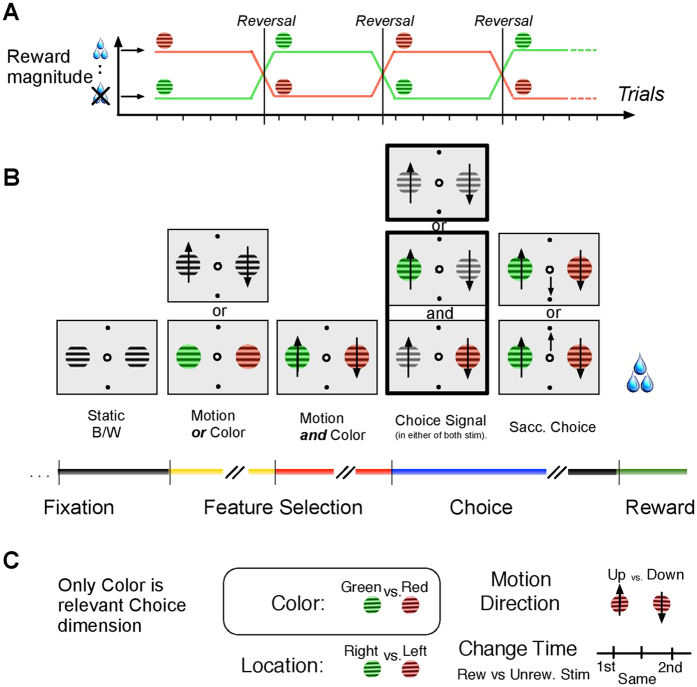
Feature-based reversal learning task. (**A**) Sketch of the reversal of colour-reward association with stimuli coloured in red (green) being associated with reward in successive blocks of trials. Colour-reward reversals were un-cued and triggered when the monkey reached a learning criterion or 50 trials. (**B**) Single trials started with fixation on a central fixation point. Two peripheral grating stimuli were shown for 0.4 sec. and either began to show movement in opposite directions, or they were coloured red/green. Following up to 0.9 sec. the feature (colour or motion) that was not present was added to the stimulus. The animal had to respond to the dimming of the stimulus with the rewarded colour. The dimming occurred either in both stimuli at the same time, or in the rewarded or the unrewarded stimulus first. Reward was provided when the animal made a saccade within 0.5 sec. after the dimming of the reward associated stimulus in the direction of motion of that stimulus. (**C**) Illustration that only colour was systematically associated with reward, while the location, motion direction or time of dimming were dimensions of the stimulus not linked to reward.

For this purpose, we report the influence of systemic Guanfacine injections on behavioural performance in a single case of a macaque monkey performing a feature-based reversal learning task (Fig. 1), in an initial 11-week dose-identifying test protocol and a subsequent 19-week behavioural testing protocol using the best working dose (for details, see Supplementary Methods).

**Figure 2:**
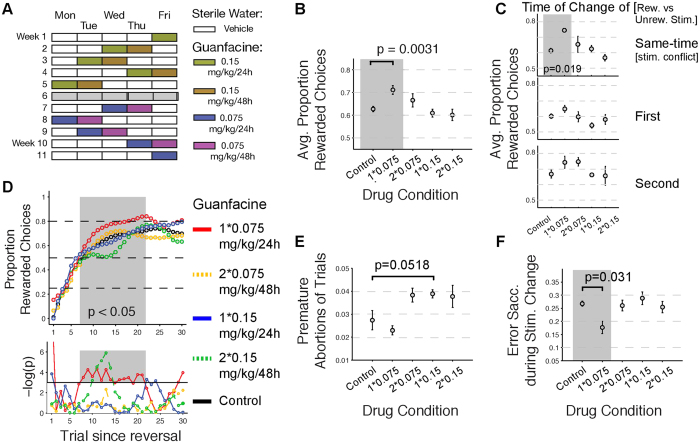
Dose-dependent improvement of reversal learning performance. (**A**) Illustration of the dose-identification protocol with blinded application of sterile water (control condition) or 0.15 mg/kg Guanfacine on two successive days in the first 5 weeks, and 0.075 mg/kg on two successive days in the last 5 weeks. No drug or vehicle was administered in week 6. (**B**) Average proportion of rewarded choices for the control and drug conditions. The bracket connects those points with statistically significant differences (Wilcoxon rank sum test). The grey background additionally highlights the significantly different pair. **(C)** Average proportion of rewarded choices separated by the time of change (top: simultaneous dimming; middle: rewarded stimulus dims first; bottom: rewarded stimulus dims second) of the rewarded versus the unrewarded stimulus for the Control and Guanfacine conditions. A significant difference was only found in the simultaneous dimming condition (top) between the control and 0.075 mg/kg/24 h condition (grey background, Wilcoxon rank sum test). (**D**) Proportion of rewarded choices across trials since the colour-reward reversal (top panel) and the evolution of p-values (as −log(p)) (bottom panels). Dark grey box highlights the trials with significantly better performance in the Guanfacine 0.075 mg/kg/24 h condition compared to the control condition (Wilcoxon rank sum test). (**E**) Proportion of trials with a premature abortion (fixation breaks) prior to onset of the stimulus colour. There were statistical trends for increased premature trial abortions with higher Guanfacine dosages. (**F**) Significant reduction of erroneous fixation breaks (e.g. toward the peripheral stimuli and without reaching the response targets) during the dimming of the stimuli with Guanfacine 0.075 mg/kg/24 h compared to the control conditions (Wilcoxon rank sum test).

During the initial 11-week drug testing protocol four doses were tested with a two doses per week schedule (see Fig. 2A), yielding for the lowest to highest dose 4, 4, 4, and 3 test sessions with 45, 31, 41, and 21 reversal blocks and 3049, 2091, 2068, 1418 trials for analysis of task performance, respectively. No drug dose had a systematic effect on the overall number of learned reversal blocks, but we found a dose-dependent effect in more fine-grained performance metrics. Firstly, the overall accuracy indexed as the overall proportion of rewarded over unrewarded choices was significantly enhanced with the 0.075 mg/kg/24 h dose compared to the control condition (Wilcoxon rank sum test, p = 0.0031), with no differences between control condition and 0.075 mg/kg/48 h, 0.15 mg/kg/24 h, and 0.15 mg/kg/48 h dose condition (all n.s.) (Fig. 2B). This enhanced overall performance improvement in the 0.075 mg/kg/24 h dose was particularly evident when calculated for the one third of trials in which the rewarded and unrewarded stimulus changed (dimmed) at the same time (Wilcoxon rank sum test, p = 0.019 for the difference of 0.075 mg/kg/24 h to control), compared to the other two third of trials in which the rewarded stimulus dimmed either before or after the unrewarded stimulus (Fig. 2C). The stimulus change (dimming) acted as go cue to elicit the choice if it occurred in the attended stimulus (see Methods). We next tested whether Guanfacine affected performance at different stages of reversal learning and found that 0.075 mg/kg/24 h of Guanfacine significantly increased performance over the control condition at trials 7 to 21 after the reversal event, i.e. during the learning period of the task and prior to asymptotic performance (see Fig. 2D, Wilcoxon rank sum test p values of p < 0.05 are shown on grey shaded area as −log(p)). In addition to this improved performance during learning with the 0.075 mg/kg/24 h dose of Guanfacine, we found reduced performance at trials 10 to 14 after colour-reward reversal for the highest dose (0.15 mg/kg/48 h) compared to the control condition (see Fig. 2D, Wilcoxon rank sum test p values of p < 0.05 are shown on grey shaded area as −log(p)). The improved performance at low dose and decreased performance at high dose are thus occurring during overlapping time periods during the learning of reversed colour-reward associations.

Analysis of the pattern of errors showed that there were similar amounts of premature fixation break errors prior to any stimulus change event with 0.075 mg/kg/24 h compared to control days, while higher doses were loosely linked with a statistical trend to higher proportions of premature fixation break errors (Wilcoxon rank sum test, p = 0.0518) (Fig. 2E). Moreover, 0.075 mg/kg/24 h Guanfacine, but no other dose, significantly reduced erroneous fixation breaks during the 0.5 sec. time period of the actual stimulus change (dimming) compared to the control condition (Wilcoxon rank sum test, p = 0.031). Further analysis of other subtypes of errors and their relation to the learning improvement were hampered by the low number of errors and the low number of testing days during the dose-testing protocol.

The previous results identified 0.075 mg/kg/24 h Guanfacine as beneficial for reversal learning performance. Higher dosages caused either no change, or were detrimental for performance and learning relative to control days. This may be due to shifts along the theoretical inverted-U plot of concentration for optimal behavioral performance that many endogenous compounds and exogenous drugs share where concentrations that are relatively too low or too high are detrimental^32^. In our study, our subject benefited from 0.075 mg/kg/24 h Guanfacine suggesting that this dose placed them closer to the peak of this inverted-U curve of optimal behavior relative to the higher dose. We tested this behaviourally beneficial dose for an extended 19-week testing protocol to test which of the behavioural performance effects would predominate and remain evident in a larger, statistically more robust dataset, and are independent of possible influences from additional injections of the drug in the same week. During this optimal dose testing protocol, the animal performed on average a similar number of reversal blocks per session in Control sessions (n: 7.96, SE: 0.38) and in Guanfacine sessions (n: 7.79, SE: 0.55) (Wilcoxon rank sum test, p = 0.4938). This provided a similar total number of reversal learning blocks for analysis in Control (n: 151 blocks) sessions and Guanfacine (n: 148 blocks) sessions with a total of 19632 trials for analysis. Across sessions, the average number of performed choices was similar for Control days (n = 332.37, SE: 14.63) and Guanfacine days (n = 334.21, SE: 18.27) (Wilcoxon rank sum test, p = 0.12). Similarly, analysis of the pattern of erroneous choices, fixation breaks indicative of distractibility, or perseverative errors indicative of inflexibility showed no prominent effect of Guanfacine compared to Control day performance during the 19-week testing period (Supplementary Results 1).

**Figure 3:**
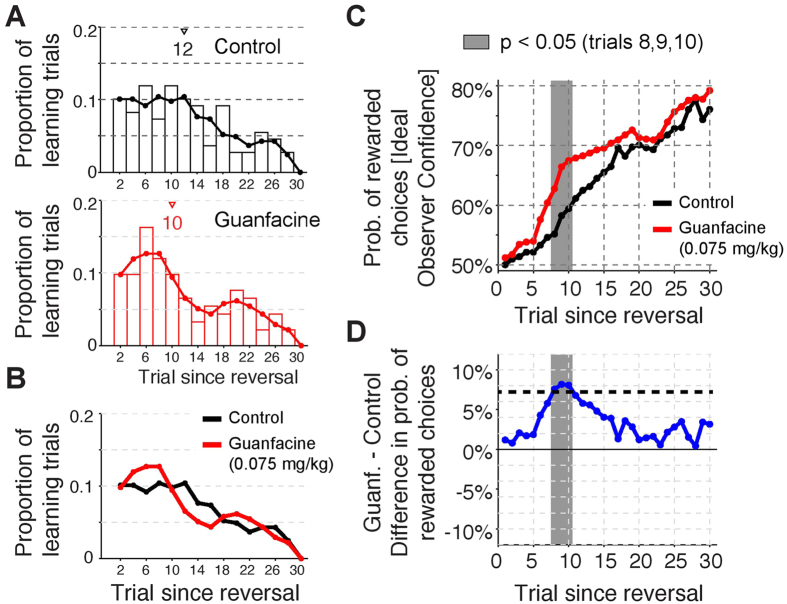
Comparison of reversal learning on Guanfacine days versus Control days. (**A**) Distribution of the proportion of trials at which learning was statistically identified across blocks in control sessions (upper panel) and in Guanfacine sessions (bottom panel). Open triangles denote the median learning trial (trial 12 for control, and trial 10 for Guanfacine sessions). (**B**) Overlay of the smoothed distribution lines from (**A**) illustrating a shift to faster learning blocks relative to slower learning blocks in the Guanfacine condition. (**C**) Median probability of rewarded choices since the reversal across all blocks that showed learning in control (black) and Guanfacine (red) sessions. The dark grey bar denotes the trial with a difference between conditions significant at p < 0.05 (dark grey), or only approaching significance at p < 0.1. (**D**) Difference of the average probability of rewarded choices in control and Guanfacine condition. Grey bars as in (**C**).

These results illustrate that Guanfacine administered once a week for 19 weeks does not simply improve overall accuracy and reduce distractibility when administered at the dose (0.075 mg/kg) that has proven to improve accuracy and reduce distractibility during the multi-dose test protocol. However, the prolonged 19-week testing could entail more specific effects on subsets of trials during reversal learning, similar to the specific improvement of behaviour during trials 7–21 since reward reversal reported above (see Fig. 2D). To test for such effects of learning we used an ideal observer approach to quantify when the succession of monkey choices indicates the actual learning of a colour-reward association since the time of colour-reward reversal (see Methods). We first verified across multiple examples that the ideal observer estimate of learning success reliably indexed reversal learning (Supplementary Fig. 1). Using the ideal observer statistics for extracting the learning trials across sessions showed that the median learning was two trials earlier on Guanfacine days (median learning: trial 10) than on Control days (median learning: trial 12) (Fig. 3A). Directly comparing the distribution of learning trials across sessions between conditions illustrates that the proportion of blocks with relatively fast learning, within 10 trials after reward reversal, was enhanced with Guanfacine, while there were less blocks with slower learning in trials 11 to 18 after reward reversal (Fig. 3B). To test statistically whether the difference between Guanfacine and Control conditions is evident at specific trials since reversal we directly compared the ideal observer confidence (which is the probability of making rewarded choices) between conditions on a trial-by-trial basis (Fig. 3C,D). We found that the probability of rewarded choices was significantly larger during 0.075 mg/kg Guanfacine than during Control days on trials 8–10 after reversal (p < 0.05, randomization test with multiple comparison correction, individual p values for trials 8–10 were: p = 0.0432, p = 0.0343, and p = 0.0359, respectively) (Fig. 3C,D). We next looked at the consistency of the behavioral enhancement of Guanfacine over all blocks in each recording day and found reliable enhancement early in the block but not late, and on average learning effects did not fluctuate across experimental sessions (see Supplementary Fig. 2 and Supplementary Results 2 and 3). In order to discern possible long-term effects of drug administration that may have an influence on overall performance we tested control session performance during the 19 week drug testing and found that learning performance remained similar for early and later control sessions (Supplementary Results 4).

### Reinforcement learning mechanisms underlying faster versus slower learning

**Figure 4:**
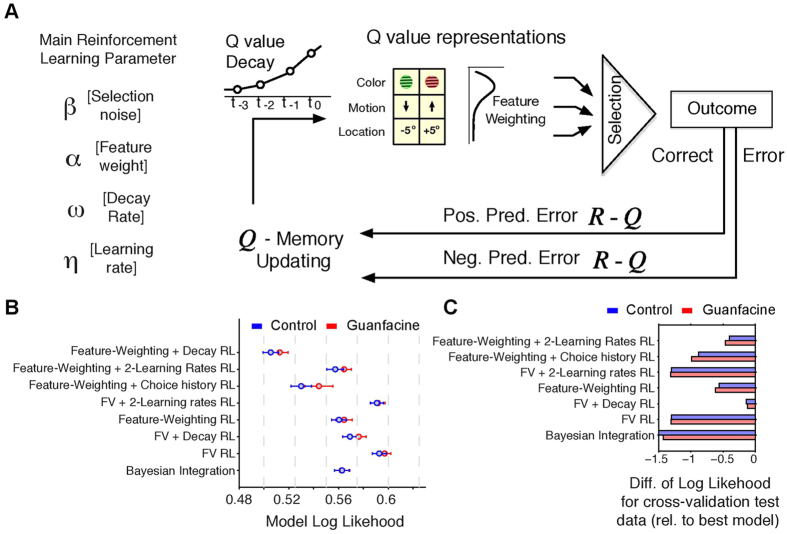
Reinforcement learning (RL) modeling of reversal learning during drug and control sessions. (**A**) Conceptual overview of the basic RL parameters (*left*) and RL mechanisms (*right*) used to account for feature based reversal learning. In the RL framework the selection of a stimulus depends on the (Q-) value prediction for the features of that stimulus (colour, location, and motion direction). Value representations can be weighted to enhance the influence of relevant features. Experiencing the outcome of stimulus selection and the saccadic choice results in a prediction error (PE), which is used to update the value prediction for future trials scaled according to a learning rate. In addition, previous studies suggest that values of non-chosen features decay according to a decay rate. (**B)** The log likelihoods for eight models described in the main text. Lower LL’s indicate better trial-by-trial prediction of the rewarded target stimulus. Error bars are STDs across 100 cross-validation training datasets. (**C**) The Feature-Weighting + Decay model provided the best LL prediction not only for the cross validation training datasets (see *B*), but also for independently predicting the 20% of reversal blocks of the test dataset. The panels show the difference in LL for the cross validation test data for all models relative to the best model. More negative values denote worse test data prediction. Red and blue points (**B**) and bars (**C**) denote LLs for the Guanfacine and control sessions, respectively.

The above results provide quantitative evidence that Guanfacine increases the proportion of blocks in which learning happens fast, i.e. within ~10 trials after reversal, relative to those blocks in which learning is slower. Such faster learning could be achieved by various underlying mechanisms that learn the reward value of stimulus features through trial-and-error (Fig. 4A). To discern which mechanism could underlie faster learning with Guanfacine we devised various learning models using either reinforcement learning (RL) of value predictions, Bayesian learning of reward probabilities, or hybrid approaches combining Bayesian learning and RL learning mechanisms^33^,^34^ (*see* Methods).

Evaluating the different models using log-likelihood based optimization and cross-validation showed that both, drug and control performance, was best predicted by the same model (Fig. 4B,C) and Supplementary Fig. 3). This *feature-weighting plus decay (FW* + *Decay) RL model* combined Bayesian- and RL- mechanisms using four parameters (*see* Fig. 4A): (1) An *α* parameter weights the relevance of stimulus features (color, location, and motion direction) in predicting high reward probabilities; (2) An *η parameter* implements the learning rate (scales the prediction error signal); (3) A *β* parameter sets the noise level of a softmax selection process for choosing one versus the other stimulus (i.e. it translates differences in value predictions into choice probabilities); (4) A decay parameter (*ω*) scales how much the reward-value predictions of non-chosen stimulus features decay over time. With these four parameters the choice patterns on both, drug and control days were predicted with highest accuracy (Fig. 4A,B). Alternative models with either the same number or fewer parameters (e.g. without the *α*, or *ω* parameter) were less accurate in predicting choices as was evident in larger model log likelihoods for the training cross validation set (Fig. 4B), worse log likelihoods for the test cross validation set (Fig. 4C), and larger deviations (Sum of Squared Errors) of the model generated choice probabilities relative to monkey proportion of choices (Supplementary Fig. 3).

To ensure that the performance of the *FW* + *Decay RL* model was not spurious due to using four parameters instead of three or two, we calculated the Akaike Information Criterion (AIC). The AIC penalizes model performance by the number of free parameters and show lowest AIC values for the model that conveys most information after considering the number of free parameters. We found that the *FW* + *Decay* RL model had the lowest AIC score (AIC: 3023.0) compared to all other models tested including a *FW (feature-weighting)* model with only three parameters lacking the decay parameter (AIC: 3331.1), and a *Feature Value Decay* RL model (*see* model 2 in methods) that included the value decay parameter, but lacked the relevance weighting of feature dimensions (AIC: 3394.6).

**Figure 5:**
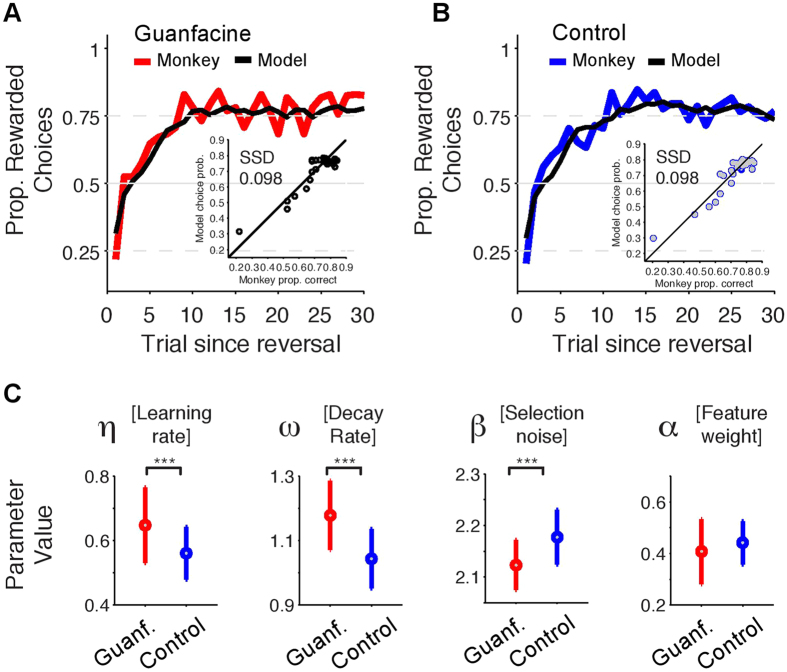
Performance and parameter values for the most-predictive RL model. (**A,B**) Proportion of rewarded choices for the monkey and model across trials since reversal in Guanfacine (**A**) and control (**B**) sessions. The model simulations are based on the best predicting *Feature-Weighting* + *Decay RL model* (see Fig. 4). The inset shows the sum of squared errors (SSD) between the proportion of correct monkey choices (*x-axis*) and the choice probability of the model across trials since reversal. (**C**) The average parameter values for n = 100 models fitted to subsets of 80% (cross-validation) reversal blocks for the Guanfacine (*red*) and control (*blue*) sessions. Errors bars denote STD. Three stars denote significance at p < 0.001 after Bonferroni correction). Guanfacine reversal performance was based on models with higher learning rate, higher decay rate and lower beta (softmax selection noise).

We next quantified whether the drug and the control performance was supported by different parameter values of the best fitting *FW* + *Decay* RL model. To this end we used the parameter values of the 80/20 cross-validation training sets as estimate for the variability of the parameter values across subsamples of the reversal blocks (Fig. 5C). We found that Guanfacine performance showed a higher learning rate (*η* drug: 0.648 STD:0.118, *η* control: 0.561 STD:0.081, t-value: 6.1, p < 0.001, after Bonferroni correction) and a stronger value-decay (*ω* drug: 1.179 STD:0.108, *ω* control: 1.043 STD:0.092, t-value: 9.529, p < 0.001, after Bonferroni correction). Notably, the beta parameter value was significantly lower for the drug condition than the control condition (*β* drug 2.12 STD:0.048 vs. *β* control 2.18 STD:0.053, t-test, t-value −7.59, p < 0.001, after Bonferroni correction). Alpha values of the optimal model for Guanfacine performance (*α* = 0.41, STD 0.126) and control performance (*α* = 0.44, STD 0.084) were not significantly different (Fig. 5D, t-test, t-value −2.18, p > 0.05, after Bonferroni correction).

**Figure 6:**
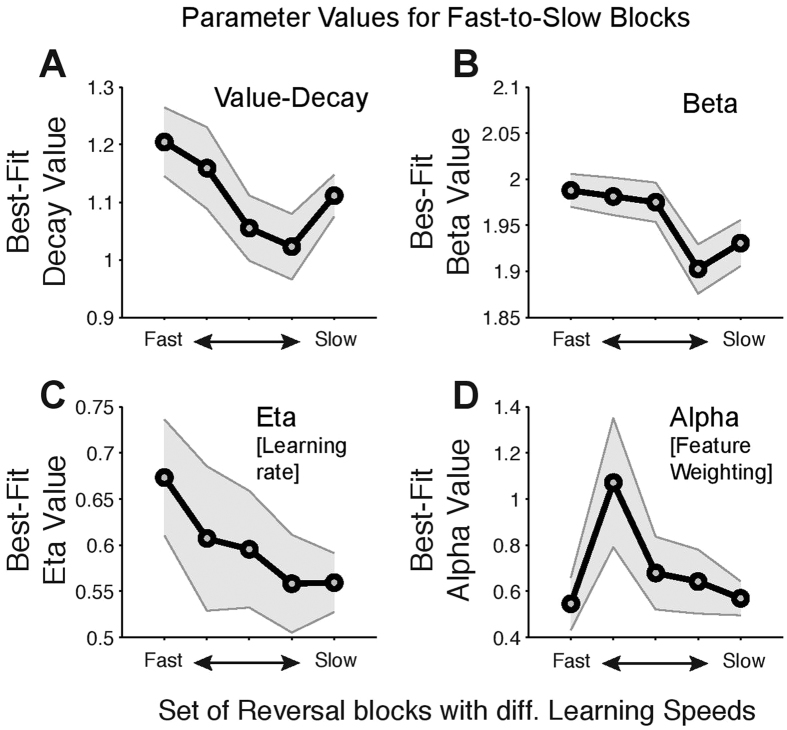
Parameter values for the Feature-Weighting + Decay RL model applied to different sets of reversal blocks showing slow and fast learning. (**A**–**D**) The value decay parameter values (*y-axis*) of the feature value decay model optimized for different sets of reversal learning blocks (*x-axis*). Bins with fast to slow reversal learning contained blocks selected according to the learning trial identified by the ideal observer statistics applied for results in Fig. 3 (*see* Methods). The five bins were 10 trials wide and slid over the data every 5 trials. The mean learning trial for each of the five bins was 6 (SE 2.6), 9.9 (SE 2.8), 14.8 (SE 3.0), 20.3 (SE 2.7), and 27.3 (SE 5.5). The panels show the optimal values for the parameters value decay (**A**), beta (**B**), eta (**C**), alpha (**D**). The error shading denote 95% confidence intervals.

We next validated that the observed parameter value space of the RL model for Guanfacine does indeed relate to the main behavioral analysis results showing faster learning with Guanfacine (*see* Fig. 3). To this end we fit the *FW* +* Decay* RL model to subsets of reversal blocks showing fast, intermediate and slow learning. This approach allows identifying the set of model parameter values that best explains the different reversal learning speeds using the actual choices of the monkey. Blocks were split into five bins according to whether the ideal observer statistics used in the behavioral analysis (*see* Fig. 3) identified learning to have occurred within trials 1–10, 5–15, 10–20, 15–25, or >20). Data from both, drug and control conditions were combined for this analysis to retain maximal number of blocks in each bin when optimizing for minimal negative log-likelihood of the model fit. We found that faster reversal learning speed is characterized by a model with higher learning rate (*η*) (Fig. 6C) and relatively larger feature-value decay (Fig. 6A). The beta parameter value remains high (*β* values >1.95) for the first three bins with relatively fast learning (with mean learning occurring at trials 6 (SE 2.6), 9.9 SE 2.8, and 14.8 (SE 3.0)) and is relatively lower in the slowest two sets of learning blocks (with mean learning occurring at trials 20.3 (SE 2.7) and 27.3 (SE 5.5)) (Fig. 6B). The *α* parameter value varies non-monotonically across the sets of learning speed (Fig. 6D). This pattern of learning speed dependent changes in parameter value space closely corresponds to the overall effect of Guanfacine on RL parameter values showing enhanced learning rate and enhanced value decay for non-chosen values (above). In summary, this analysis establishes a link between the behavioral analysis showing faster learning with Guanfacine and the RL model fitting approach showing variations of parameter values best explaining the learning behavior under Guanfacine.

**Figure 7:**
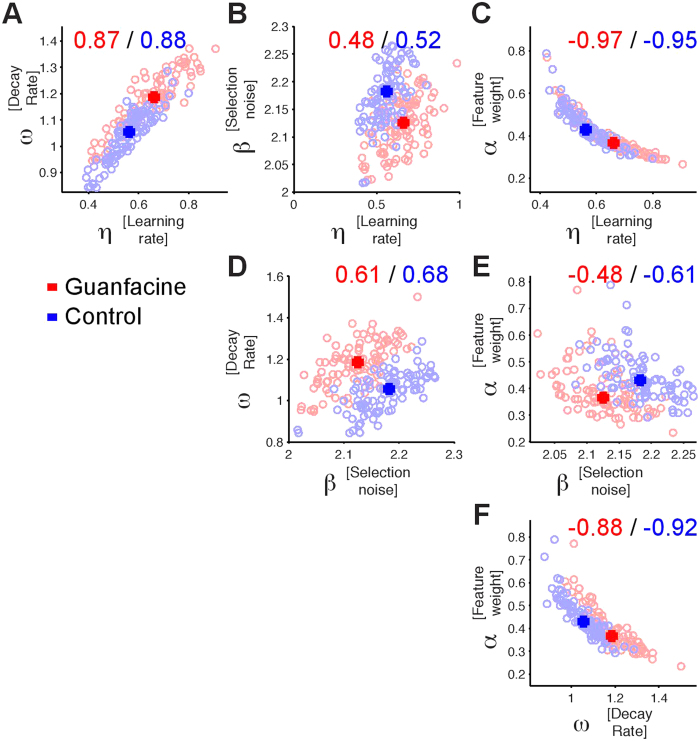
Relation of model parameters underlying reversal performance. (**A**–**C**) Changes in learning rate (*x-axis*) across n = 100 cross validation training models are positively correlated with value decay (**A**) and beta selection noise (**B**), and negatively correlated with feature weighting (**C**). Red and blue numbers denote the correlation coefficient for Guanfacine (*red*) and control (*blue*) data points. (**D–F**) Same format as (**A–C**) but showing scatterplots of the correlation between beta selection noise, decay rate and feature weighting.

The modeling results showed that Guanfacine performance is linked to changes of more than one RL parameter raising the question on whether the model parameters are affected independently, or whether they co-vary to account for the faster learning performance. We tested this question by correlating the values of pairs of parameters from the optimal *FW* + *Decay* RL model of the n = 100 subsampled datasets (from the 80/20 training cross validation runs). This analysis showed significant correlations among all parameter pairs (Fig. 7). Larger learning rates were associated with larger value decay (*ω*) and larger *β* values (Fig. 7A,B,D), while larger feature-weighting (*α*) was associated with lower learning rate, *β*, and feature value decay (*ω*) (Fig. 7C,E,F). These findings corroborate the suggestion that compared to control performance Guanfacine modulates the values of more than one parameter and hence acts on multiple RL mechanisms.

## Discussion

Using behavioral analysis and computational modeling of a single subject’s performance, we found that Guanfacine can enhance specific reinforcement learning mechanisms supporting reversal learning. Initial dose testing over a short time period showed that this concentration was capable of enhancing overall performance and reducing distractibility from simultaneous luminance changes occurring in non-relevant and relevant stimuli (Fig. 2). Higher doses of Guanfacine did not improve performance and, when given for two successive days, significantly reduced performance. The second, longer experimental phase similarly showed improved learning effects with the best Guanfacine concentration, becoming evident in reliably faster reversal blocks with increased learning success within the first 10 trials in the drug condition compared to the control condition. This enhancement in reversal learning was evident in the absence of changes in other performance measures such as (1) overall motivation to perform the task (number and length of performed trials), (2) attentional interference control (influences of distractors on accuracy), (3) impulsivity (proportion of premature responses), or (4) perseveration tendencies (repetitions of unrewarded responses). Analysis of the reinforcement learning mechanisms identified one model that best accounted for both, drug and control performance. The model parameter values suggested that the Guanfacine effect on fast learning is not achieved by modifying a single learning parameter. Rather, our findings suggest that Guanfacine may shift values in the parameter space of the reinforcement learning towards higher learning rates and more pronounced decaying of the value of non-chosen stimulus features. Both of these parameters showed higher values for faster as opposed to slower learning blocks validating that the Guanfacine effect on learning improvement could originate from larger learning rates and stronger decay of feature values of non-chosen stimuli. In summary, these findings indicate that Guanfacine facilitated behavioural flexibility at the subject-specific drug concentration in a task requiring selective attention to the value of stimulus features and their reward outcomes over multiple reversals of stimulus relevance per daily session. These results may have implications for the clinical usage of Guanfacine for treating ADHD and multiple other conditions characterized by learning disabilities, attention deficits, or impaired behavioural flexibility^1^,^32^.

### Alpha 2A noradrenergic action supports multiple routes to behavioural flexibility

The primary behavioural signature of Guanfacine in our task is an enhanced reliability to learn from trial-and-error during the first ten trials after reversal. This reversal was un-cued and hence became apparent to the subject by experiencing unexpected erroneous outcomes after attending a now non-rewarded (in the current block), but previously rewarded (in previous block) stimulus colour. Guanfacine enhanced the likelihood to use these erroneous outcomes and to increase more quickly the ideal observer confidence that a new colour has become rewarded. This behavioural pattern parsimoniously can be described to reflect enhanced flexibility to adjust to changing reward contingencies in the task environment, e.g. by identifying how a current task situation (or ‘state’) differs to a previous situation^35^ and by updating the internal beliefs about feature-reward contingencies^36^,^37^. Such update-specific action of noreprinephrine has been inferred in previous human studies from putatively norepinephrine mediated pupil dilation changes specifically during task epochs that required an update of beliefs to better predict future events^36^ and to better predict future saccade target locations^37^. These studies support the interpretation that the main effect of Guanfacine in our task was to facilitate the updating of color-reward contingencies during the learning process. According to this interpretation, Guanfacine increases endogenous control over stimulus selection during periods when changing environmental reward contingencies call for adjusting beliefs and behavior^38^,^39^. There are multiple routes how such a higher level effect could be implemented and supported by Guanfacine action. For example, to improve flexibility in responding to environmental changes can be achieved by (1) enhanced attentiveness and control of interference from distractors, (2) from preventing perseverations and habitual responding, (3) from increased vigilance and arousal, (4) from increasing the representations about which features are relevant in a working memory that persist across trials, or (5) from lowering impulsive response tendencies.

Among these many possibilities of Guanfacine action, the best understood effect is enhanced working memory. Previous nonhuman primate studies have documented improved working memory performance in delayed response, delayed match-to-sample, and delayed non-match-to-sample tasks, requiring short-term maintenance of stimulus locations and object identities^1^. Young and aged nonhuman primates tolerate increased delays at subject specific Guanfacine doses ranging from as low as 0.00015 to 0.5 mg/kg (see Table 1). This working memory benefit has been traced back to Guanfacine induced increases in spatially specific delay firing in lateral PFC^4^,^5^. This prefrontal effect of a2A-NAR activation is well explained by blocking cAMP signaling and concomitant increases in NMDA conductance at the spines of pyramidal cells^4^,^5^,^40^.

These insights reveal that a2A-NAR activation specifically increases task relevant representations in the PFC, making it likely that such an effect contributes to the behavioural improvements that we report. This contribution would be plausible if Guanfacine would not only increase the representation of stimulus location or prospective saccade location that would explain previous studies’ effects, but if it would enhance the representation of colour-reward conjunctions irrespective of the location or saccadic action plan. An enhanced working memory of which stimulus features are currently task relevant (rewarded) could reduce the need for explorative choices and increase the confidence in trial-by-trial selections^9^. These effects could indirectly become visible in the enhanced decay, i.e. active suppression, of values from non-chosen stimuli, and thus could culminate in faster learning rates as we observed in the RL model. However, this account predicts that Guanfacine should not only improve the initial reversal learning, but should increase overall performance accuracy. We did not find this effect, suggesting that Guanfacine’s primary behavioral effects are on alternate mechanisms.

One alternate mechanism that has been associated with phasic noradrenergic activation is enhanced control of interference, a main pre-requisite for flexible behaviour that strives towards achieving a goal irrespective of distractions^8^,^41^,^42^. Evidence for this suggestion derives from rodent studies^43^ (see also ref. 44) and from human studies describing reduced scores of distractibility in ADHD patients treated with Guanfacine^27^. Intriguingly, we observed enhanced focusing in our task during the three sessions of drug testing at the optimal 0.075 mg/kg dose with enhanced performance during trials with an enhanced stimulus conflict, i.e. when targets and distractors dimmed simultaneously rather than at separate times (Fig. 2D). However, this effect did not retain across the nineteen-week behavioural testing sessions that commenced after the dose testing at the same concentration (0.075 mg/kg), which notably is in the same range (0.05–0.12 mg/kg) proposed to be effective for extended release medication in ADHD^45^,^46^. This suggests that the influence of a2A-NAR activation on interference control at the dose tested is not a primary effect in healthy brains and may only appear when the attention system is compromised. This conclusion resonates with the difficulty to observe Guanfacine effects in healthy humans on attentional set shifting tasks^19^, and with a previous Guanfacine study in aged monkeys showing an improved performance to select rewarded over non-rewarded objects that were reversed once over the course of a week^47^. Taken these lines of evidence together suggests that Guanfacine’s influences on interference control do not explain our main findings in this study, but rather may be unmasked in aging or disease states when the strength of target representations is compromised.

Another contribution to improved learning in our task could be an increase in vigilance, or so-called ‘scanning attentiveness’ that has been hypothesized to be a main route for noradrenergic action^30^. This aspect is particularly important for our task, because it required repeated reward reversals in a single experimental session that continued for an extended duration (≥55 min) until the subject self-terminated working. We can rule out that Guanfacine simply prolonged vigilance, as we did neither observe longer performance, nor a change in the average number of learning blocks per session, and learning speed at the end of experimental sessions was similar for control and Guanfacine days (Supplementary Fig. 3).

### Reinforcement learning modeling of behavioural drug effects advances computational psychiatry

Our study tested eight reinforcement learning models to recover the possible learning mechanisms underlying the observed behavioural drug effect on reversal learning and arrived at the same model, the *feature-weighting* + *decay (FW* + *decay*) RL model, to account for both, drug and control reversal learning. We found that this model provided the best independent prediction for test-data during cross-validation (Fig. 4C), and allowed the generation of choices that closely resembled the subject’s choice patterns (Fig. 5A,B). Moreover, we found that the two model parameters that characterized faster learning during Guanfacine than control sessions, were directly linked to the results from the model-free behavioral analysis results that showed faster learning with Guanfacine. This observation provides evidence that the model captures some fundamental learning principles underlying task performance, supporting the notion that such modeling will be pivotal to understand the working mechanisms of behavioural neuromodulation^48^ and, more generally, to approach better testable theories of cognitive dysfunction in the new field of computational psychiatry^49^. We see our RL modeling as an early starting point to approach individualized, subject-specific characterization of cognitive profiles that are called upon in currently developed neuropsychiatric research frameworks (e.g. ref. 50). This framework accepts that there will be individual differences in learning and choice behaviors that call upon the characterization of what could be called a subject-specific drug effect on the parameter space of the underlying learning and attention systems. We embrace this approach with this single-case monkey study, but note the necessity that large samples of subjects are needed to arrive at conclusions that hold at the population level. We expect that future studies will extend this modeling endeavor, for example, by separating learning rates from sensitivity to reward per se^51^, dissociating value-based prediction processes from value-independent biases of subjects (e.g. ref. 34), and estimating the type of state representation that best explains value predictions and choices in various tasks employed^33^,^52^,^53^.

In conclusion, the results presented here illustrate how a computational approach links the influence of alpha 2AR activation to variations of formally defined reinforcement learning mechanisms. We expect that such a linkage will be pivotal to advance our understanding of higher-order cognitive phenomena such as distractibility and flexible adjustments of attentional sets following feedback^29^. Firstly, these phenomena closely relate to fundamental RL mechanisms and thus can be captured with a common terminology in a unifying theoretical framework^28^,^54^,^55^. Such common terminology will facilitate comparison of results between studies, task paradigms, study subjects and between species. Secondly, the power to predict single-subject drug effects on behavior bears enormous potential for individualizing treatments in psychiatry. For example, recent studies have shown that knowledge of the formal model and parameter values best describing individual subjects, provide hints to the underlying cognitive weaknesses that can be targeted with drugs affecting those specific weaknesses (e.g. refs 56,57). Thirdly, we believe that a computational framework as we applied here may prove to be essential to identifying the neuronal mechanisms underlying the neurochemistry of higher cognitive functions. A main reason for this potential is that formal Bayesian and RL models provide essential information about hidden variables that account for variations in behaviour not captured by raw performance data (e.g. refs 58,59).

## Methods

### Subject and apparatus

Data was collected from a 9 year-old male rhesus macaque (*Macaca mulatta*). All animal care and experimental protocols were approved by the York University Animal Care Committee and were in accordance with the Canadian Council on Animal Care guidelines. Eye positions were monitored using a video-based eye-tracking system (Eyelink 1000 Osgoode, Ontario, Canada, 500 Hz sampling rate), and calibrated prior to each experiment to a 9-point fixation pattern. During the experiments, stimulus presentation, eye position monitoring, and reward delivery were controlled via MonkeyLogic (open-source software http://www.monkeylogic.net). Reward was delivered as liquid drops from a sipper tube in front of the monkey’s mouth and controlled from an air-pressured mechanical valve system (Neuronitek, London, Ontario, Canada). To ensure the monkey’s motivation, fluid intake was controlled during training and experimental sessions; unrestricted access to monkey chow was available. The experiments proceeded in a dark experimental booth with the animal sitting in a custom made primate chair with the eyes 65 cm away from a 21′ LCD monitor refreshed at 85 Hz.

### Behavioural paradigm

The monkey performed a variant of a feature-based reversal learning task^47^ that required covert spatial attention to one of the two stimuli, the identity of which depended on the current colour-reward association. To obtain reward, an up-/downward saccade had to be performed to the motion direction of the attended stimulus, which was varied independently from the colour of the stimuli. The colour-reward associations were reversed in an un-cued manner between blocks of trials with constant colour-reward association (Fig. 1A). By separating the location of attention from the location of the saccadic response, this task allowed studying visual attention functions independent of motor intention related processes during reversal learning. Each trial started with the appearance of a grey central fixation point, which the monkey had to fixate. After 0.5–0.9 s, two black/white drifting gratings appeared to the left and right of the central fixation point (Fig. 1B). Following another 0.4 s the two stimulus gratings either changed colour to black/green and black/red, or started moving in opposite directions up and down, followed after 0.5–0.9 s by the onset of the second stimulus feature that had not been presented so far, i.e. if after 0.4 s the stimulus gratings changed colour then after another 0.5–0.9 s they started moving in opposite directions or vice versa. After 0.4–1 s either the red and green stimulus dimmed simultaneously for 0.3 s or they dimmed separated by 0.55 s, whereby either the red or green stimulus could dim first. The dimming represented the go-cue to make a saccade to one of two response targets displayed above and below the central fixation point (Fig. 1B). Please note that the monkey needed to keep central fixation until this dimming event occurred. A saccadic response following the dimming was only rewarded if it was made to the response target that corresponded to the movement direction of the stimulus with the colour that was associated with reward in the current block of trials, i.e. if the red stimulus was the currently rewarded target and was moving upward, a saccade had to be made to the upper response target at the time the red stimulus dimmed. A saccadic response was not rewarded if it was made to the response target that corresponded to the movement direction of the stimulus with the non-reward associated colour. A correct response was followed by 0.33 ml of water delivered to the monkey’s mouth. Across trials within a block, the colour-reward association remained constant for 30 to a maximum of 50 trials. Performance of 90% rewarded trials (calculated as running average over the last 12 trials) automatically induced a block change. The block change was un-cued, requiring the subject to use the reward outcome they received to learn when the colour-reward association was reversed in order to covertly select the stimulus with the rewarded colour. In contrast to colour, other stimulus features (motion direction or stimulus location) were only randomly related to reward outcome (Fig. 1C).

To ensure the deployment of covert attentional stimulus selection we dimmed the rewarded stimulus only after the dimming of the unrewarded stimulus in one third of the trials (requiring the attentional filtering of the unrewarded stimulus). In another third of trials the rewarded and unrewarded stimulus dimmed at the same time, which probed the animal to focus attention prior to the dimming to resolve the stimulus conflict from the simultaneous dimming. In the remaining third of trials the rewarded stimulus dimmed prior to the un-rewarded stimulus. This timing regime ensured that first, second and same-time dimming of the rewarded versus unrewarded stimulus occurred unpredictably for the monkey. Saccadic responses had to be initialized within 0.5 s after dimming onset to be considered a choice (rewarded or non-rewarded). All other saccadic responses, e.g. towards the peripheral stimuli, were considered non-choice errors.

### Experimental procedures for dose identification testing protocol

In each experimental session the monkey was given the opportunity to perform the task for a minimum of 55 minutes after which, if he chose to continue, he could do so indefinitely. However, if he chose to stop working, he was given an additional 5 minutes before the session was stopped by the experimenter. If a trial was successfully completed within these 5 minutes, the timer would re-set and allow him another 5 minutes before the daily behavioural session was ended. This procedure led to an average working duration of 68.7 minutes (SE 0.21).

For treatment sessions, the monkey received an intramuscular (IM) administration of Guanfacine (Guanfacine hydrochloride, Sigma-Aldrich, St. Louis, MO), or an IM injection of sterile water at about 2.5 h before the first trial of the experimental session (across sessions the average time was 150.8 minutes (SE: 0.88)). This time frame is similar to previous studies that have shown significant effects of Guanfacine on cognition in young and aged monkeys^14^,^30^. Immediately prior to IM administration, Guanfacine was mixed with sterile water as vehicle; the total injection volume was 0.1 ml. Doses of Guanfacine investigated were 0.3, 0.15 and 0.075 mg/kg. 0.3 mg/kg was used in only two sessions and was discarded because it caused increased fixation breaks of the animal during the trial, which ruled out overall positive effects at that dose. Doses were chosen as previous studies have found significant enhancements in cognition with similar doses of Guanfacine (e.g. ref. 14). We performed a meta-survey of all available nonhuman primate studies that used Guanfacine to evaluate the dose range and expected cognitive effects in our study (please see Table 1).

To identify the dose of Guanfacine that is behaviourally beneficial we applied an efficient 11-week dose identification testing protocol that allowed us to discern drug effects of the same dosage given on two consecutive days (Fig. 2A). All other days prior or following treatment days were control days with control injections. Treatment days were shifted randomly weekly and could occur on any two consecutive days during the week, thereby balancing the drug injection weekdays across the testing period. During the entire dose identifying protocol, drug administration was blinded, hence the experimenter did not know whether a given day was a treatment or control day. All experimental sessions were conducted at the same time of day. Prior to this experiment, the monkey had not received any Guanfacine, or any other catecholaminergic drugs, in an experimental setting.

### Experimental procedures for optimal dose testing protocol

Following the 11-week dose testing protocol and a 4-week washout period we tested the influence of the dose that resulted in improved behavioural learning during the dose identifying test protocol. To this end we applied control injections on one day a week and Guanfacine (0.075 mg/kg) injections on another day of the week 75–120 min prior to commencing behavioural testing of the animal. Injection procedures were identical to those described above. This optimal-dose testing protocol provided 19 control sessions and 19 sessions with Guanfacine 0.075 mg/kg. Behavioural task, fluid control regimes for the animal, and reward schedules were identical to the previous testing protocol.

### Behavioural analysis of learning trials

Analysis was performed with custom MATLAB code (Mathworks, Natick, MA), utilizing functionality from the open-source fieldtrip toolbox (http://www.ru.nl/fcdonders/fieldtrip/). To identify at which trial during a block the monkey showed statistically reliable learning we analyzed the monkeys’ trial-by-trial choice dynamics using the state–space framework introduced by Smith and Brown^60^ (see ref. 32, Supplementary Methods and Supplementary Fig. 1 for examples).

### Testing for trial-by-trial differences of the probability of rewarded choices

To test whether the probability of rewarded choices differed between drug and control conditions in specific trials following the first trial after the reversal we applied permutation statistics. In particular, we tested the null hypothesis that the probability of rewarded choices at individual trials since reversal is the same in drug and control conditions. To test this hypothesis we extracted the average (median) probability of rewarded choices for each trial since the reversal until trial 30 across blocks of the Guanfacine condition and across blocks of the control condition. We used the difference in the average probability of rewarded choices between conditions for each trial since reversal as test statistics in a randomization test that corrected for multiple comparisons across trials. For the randomization procedure, we extracted the difference in the average probability of rewarded choices for each trial since reversal n = 1000 times with randomly assigned condition labels. To correct for multiple comparisons, we pooled the random distributions across trials and calculated the 95% threshold value (the 28.500’s of 30.000 values) of the difference in the probability of rewarded choices that would be obtained when the condition labels were unknown. We then compared the observed differences between Guanfacine and Control conditions in trials 1 to 30 to the 95% threshold value. If the observed difference at any trial in the block exceeds the threshold value it can be inferred that reward probability is significantly higher in the Guanfacine compared to the control condition at p < 0.05. This randomization procedure prevents multiple comparison correction by calculating a single threshold value across trials.

### Testing for the consistency of learning differences across blocks within sessions

The effect of Guanfacine on learning could be consistent within an experimental session, or it could increase or decrease across blocks within a session. We tested for the consistency of learning effects by first extracting the learning trials for all blocks performed during behavioural test sessions using the ideal observer estimate of learning described above^61^. We then calculated the average (median) learning trial across four successive blocks starting with the first four blocks since reversal and stepping from the first to the eights block of a session. For each set of blocks we calculated the median learning trial in the Guanfacine sessions and in the control sessions. This procedure provided the average learning trial for each block relative to the first block in a session. We then repeated the procedure, but starting from the last block in a session and going backwards, averaging the learning trials in the last four blocks, the second to last four blocks, etc. until the seventh to last block. This procedure provided an estimate of the change in median learning trials relative to the end of the session. This was done to account for the variability in the number of blocks completed in any given experimental session.

To test whether the average learning trials were consistently earlier or later in the Guanfacine condition relative to the control condition we used a randomization procedure. For this purpose we used as test statistics the proportion of blocks with an average learning trial that was earlier in the Guanfacine condition than in the control condition. This test statistics included eight average learning trials since reversal and seven average learning trials since the last block in a session (see above and Fig. 5). We then tested the null hypothesis that the drug condition label (Guanfacine or Control) has no effect on the proportion of earlier learning trials. To this end we computed n = 1000 times the proportion of blocks with an earlier learning trial in a random condition A relative to condition B with random assignment of Guanfacine and Control blocks to conditions A and B. We then calculated the p-value as 1 minus the proportion within which the truly observed proportion of blocks with earlier learning trials in the Guanfacine condition relative to the control condition exceeded the proportion of earlier learning in the n = 1000 random distribution. Guanfacine would consistently have resulted in earlier learning trials across blocks when the true observed learning trial was earlier than in control conditions in >95% of the random distribution that was blind to the condition label.

### Reinforcement learning modeling

In order to infer possible learning mechanisms underlying the behavioral drug effects we tested various computational models using reinforcement learning and Bayesian learning principles following an approach and terminology from Niv and Wilson and colleagues^33^,^61^. These models aim to find the potential variables that can predict which of the two stimuli the subject picks on a given trial given the history of stimuli, rewards, and choices on past trials up to trial t, which will be denoted by 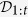. We assume that the subject represents the past trials’ data as a set of values, rather than keep the entire past in memory, that is, there are quantities that can act as so called sufficient statistics. Models are comprised of specifying whether features (color, motion, location), feature values (colour A, colour B, downward motion, upward motion, left, right), or stimuli (combinations of feature values) are assigned a value, and how this value is updated following a new choice and its outcome (i.e. whether a reward was received or not).

The *first* model, Feature-Value Reinforcement Learning (*FV RL*), assigns values to feature values that define each stimulus. There are three features in each of the two stimuli, the location (left (L) versus right (R)), the direction of motion (up (U) or down (D)) and the color (1 or 2). Across the whole experiment there are only two different colors in each presented stimulus configuration, hence we indicate them just as 1 and 2. This yields six different feature values: L, R, U, D, 1, 2, which we will label with the indices 1 to 6, the corresponding value is thus V_i_. A presented stimulus has a value for each of three features, and thus possesses 3 feature value combinations (FVCs), the other stimulus has the remainder of the FVCs. All the FVCs corresponding to the chosen stimulus are updated, because each of them in principle could be a target that was rewarded, which of the three FVCs is the target can only be disambiguated across the presentation of multiple informative stimulus configurations. After receiving an outcome *R* (1 if rewarded, 0 if non rewarded) the value update is done according to


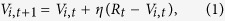


for all FVCs *i* that belong to the stimulus. This equation ensures that when there is a difference between the received reward and the expected (predicted) reward, the value gets updated to get closer to the received reward–implementing the delta rule of classical prediction error learning, with η representing the learning rate. When η = 1, the new value is set to R_t_, when η exceeds 2, the update becomes unstable, as it can grow without bound.

The choice C_t_ (which stimulus) is made by a softmax rule according to the sum of values of each FVC that belongs to the stimulus. We indicate the stimulus by the index j and the set of feature values that belong to it by s_j_.


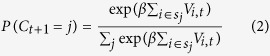


The *second* model, Feature-Value plus Decay Reinforcement Learning (*FV* + *Decay RL*), is an extension of the first model, and includes in addition a decay constant, which reduces the value of the FVCs of the stimuli that were not chosen. The feature values belonging to the chosen stimulus are updated according to eq. 1. The feature values *i* of the non-chosen stimulus decay according to


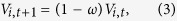


The decay parameter is denoted by ω. The choice is made as before (eq. 2).

The *third* model, Feature-Value with 2-Learning Rate Reinforcement Learning (*FV* + *2 Eta RL*), is also an extension of the first model, in that it includes two different learning rates, one for when the choice is rewarded (η_1_) and the other (η_0_) for when it is not. The value update proceeds according to


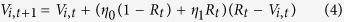


The choice again is made as described before (eq. 2).

### Bayesian learning modeling

The remaining models have a Bayesian component, which we introduce here and which has been described in detail elsewhere^33^. The learning goal is to choose the stimulus that gives a reward, hence the one that has the target feature value (color 1 or 2). The information provided in each trial can be accumulated across trials by using Bayes’ rule. This starts from the probability of obtaining a reward R_t_ as a function of the presented stimulus S_t_ and the choice C_t_ made assuming the target feature value combination is f: *p(R*_*t*_|*C*_*t*_, *f*) = *p*_*r*_*R*_*t*_ + (1 − *p*_*r*_)(1 − *R*_*t*_) and thus that the chosen stimulus S_Ct_ contains f. The expression tells us that the probability for getting reward (R_t_ = 1) is p_r_ and for getting no reward (R_t_ = 0) is (1 − p_r_). When the chosen stimulus S_Ct_ does not contain f, *p(R*_*t*_|*C*_*t*_, *f*) = *p*_*n*_*R*_*t*_ + (1 − *p*_*n*_)(1 − *R*_*t*_). We can combine these two expressions into one by defining S_Ct_(f) = 1, when it contains feature f, and zero otherwise yielding


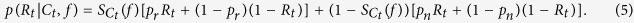


The calculations simplify further when choosing *p*_*n*_ = 1 − *p*_*r*_. What we are interested in is 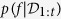, and aim to express it iteratively in terms of 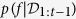. We start the iteration from a uniform initial distribution representing the lack of knowledge about the target. Each trial gives independent information, hence we can write


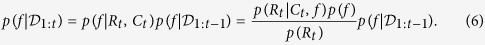


The expression depends only on f and factors that do not depend on f, such as p(R_t_), will be taken into account as a consequence of normalization of this probability distribution across f. On trial t, when ignoring the past, target f could be anything, hence p(f) is constant, we thus obtain:


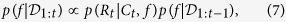


where after each update we need to normalize this distribution again.

The *fourth* model BI (*Bayesian integration*), adapted from previous reports^33^,^61^, uses as a value the probability of reward on a new trial, as a function of the choice (still to be made), given the past data:


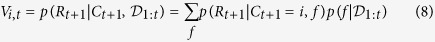


The choice is then made in the same way as before using a Boltzman function with parameter β:


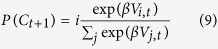


We noticed that Bayesian updates are much faster than expected from the subjects’ choices (see e.g. Supplementary Fig. 3A), hence we kept p_r_ as a parameter. In the experimental setup the subject will receive a reward when it makes the correct choice, hence p_r_ = 1, but here we take p_r_ = 0.99 < 1 in which case the Bayesian integration is slower.

### Hybrid Bayesian-Reinforcement learning modeling

The *fifth* model, Bayesian Feature-Weighting Reinforcement Learning (*FW RL*) combines the Bayesian inference of the target f via 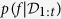, with values for all feature value combinations. We introduce a new notation to properly specify the model: f normally takes 6 values, now we use f_d_, where d represents the dimension or feature (1: location; 2: direction of motion, 3: color) and for each d, f_d_, takes two values 1 and 2. For instance, f_3_ = 1 indicates the first color. We can then calculate the probability for the target to have feature d, 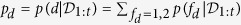. This defines a feature dimension weight 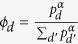, with exponent α and normalized to yield a sum across dimensions equal to one. The predicted reward value of a feature value is then denoted by 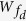 and the value of stimulus i is given by the sum across all feature values that are part of the stimulus


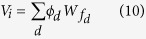


The choice is then again given by a Boltzmann function


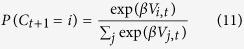


In addition to the Bayesian update of the feature weights, the values of each Feature value of the chosen stimulus is updated as well, with a prediction error that is the difference between the rewarded and the calculated value of the chosen object, rather than the value of the feature value:


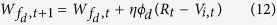


The *sixth* model, Bayesian Feature Weighting plus Choice History Reinforcement Learning (*FW+Choice History RL*), extends the fifth model by an influence of choice history that is independent of reward history and was found in a previous experiment to be a superior model^34^. Choice history is included in a two-step selection process. First, it calculates the values V_i_ and choice probabilities *P*_*j*_ = *P(C*_*t*+1_ = *j*). as before and makes a stochastic choice j. It then compares whether the so chosen stimulus has the same color as the previous choice. If this is the case the choice is accepted, otherwise it will be accepted with probability


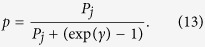


If it is not accepted the other stimulus will be chosen instead, which is the one that matches the previously chosen color.

The *seventh* model, Bayesian Feature Weighting + 2 Learning Rates Reinforcement Learning (*FW+2 Eta RL*), combine feature weighting (model 5) with the updating with two different η-values (model 3). The only change with respect to the procedure outlined for model 5 is the update of the values for each feature value:


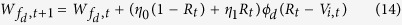


The *eight* model, Bayesian Feature Weighting + Decay Reinforcement Learning (*FW+Decay RL*) combines feature weighting (model 5) with the update with decay (model 2). The feature value belonging to the chosen stimulus are updated according to 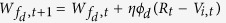, whereas those belonging to the non-chosen object are updated according to 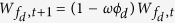.

### Model optimization, evaluation and comparison

The RL models were optimized by minimizing the negative log likelihood over all trials using up to 20 iterations of the simplex optimization method (matlab function fminsearch) followed by fminunc which constructs derivative information. We used a 80%/20% (training dataset/test dataset) cross-validation procedure repeated for n = 100 times for each of the eight models. Each of the hundred cross-validations per model optimizes the model parameters on the training dataset. We then quantified the log-likelihood of the independent test dataset given the training datasets optimal parameter values (see Fig. 4C). We used the variability of the training datasets’ optimal parameter values to evaluate their standard deviation (see Fig. 4B), and to evaluate how the values of different model parameters co-vary (see Fig. 7).

To compare RL models with different numbers of free parameters we calculated the Akaike Information Criterion (AIC) for each best-fit model as [*2k* − *2ln(L*)] with *k* reflecting the number of free parameters and *L* the maximum likelihood value of the model. Lower AIC values indicate a better model fit after penalizing for the number of free parameters used for fitting the respective model.

## Additional Information

**How to cite this article**: Hassani, S. A. *et al*. A computational psychiatry approach identifies how alpha-2A noradrenergic agonist Guanfacine affects feature-based reinforcement learning in the macaque. *Sci. Rep.* **7**, 40606; doi: 10.1038/srep40606 (2017).

**Publisher's note:** Springer Nature remains neutral with regard to jurisdictional claims in published maps and institutional affiliations.

## Supplementary Material

Supplementary Information

## Figures and Tables

**Table 1:**
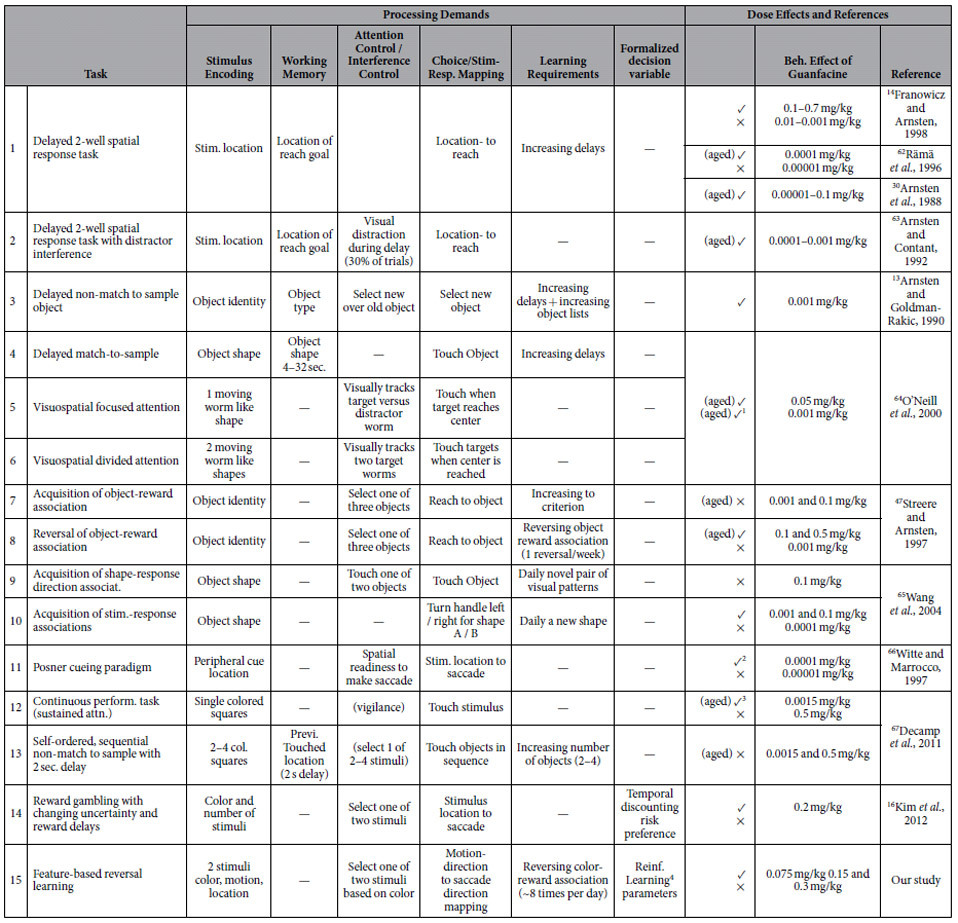
Meta-survey of cognitive effects from systemic Guanfacine administration in non-human primates.

Columns indicate the cognitive subfunctions, the dosages, and the obtained effect (tick mark indicates statistical significance, cross indicates lack of significance), and the study reporting the effect. Rows indicate the experimental manipulation tested during systemic drug administration. Note that some studies use different tasks and different dosages of Guanfacine.

^1^Low dose improved accuracy in one of two animals.

^2^No effects on accuracy and cue validity, and opposite signs of altering effect with increased and decreased reaction times in each monkey.

^3^Performance improvement evident in less omission errors, but accuracy (commission erros) was unaffected.

^4^Reinforcement learning parameters (learning rate, inverse temperature selection parameter) were not individually significant, but contributed to improved learning.

## References

[b1] Arnsten A. F., Wang M. J. & Paspalas C. D. Neuromodulation of thought: flexibilities and vulnerabilities in prefrontal cortical network synapses. Neuron 76, 223–239 (2012). 23040817 10.1016/j.neuron.2012.08.038 PMC3488343

[b2] Arnsten A. F. & Dudley A. G. Methylphenidate improves prefrontal cortical cognitive function through alpha2 adrenoceptor and dopamine D1 receptor actions: Relevance to therapeutic effects in Attention Deficit Hyperactivity Disorder. Behav Brain Funct 1, 2 (2005). 15916700 10.1186/1744-9081-1-2 PMC1143775

[b3] Clark K. L. & Noudoost B. The role of prefrontal catecholamines in attention and working memory. Front Neural Circuits 8, 33 (2014). 24782714 10.3389/fncir.2014.00033 PMC3986539

[b4] Wang M. . Neuronal basis of age-related working memory decline. Nature 476, 210–213 (2011). 21796118 10.1038/nature10243 PMC3193794

[b5] Wang M. . Alpha2A-adrenoceptors strengthen working memory networks by inhibiting cAMP-HCN channel signaling in prefrontal cortex. Cell 129, 397–410 (2007). 17448997 10.1016/j.cell.2007.03.015

[b6] Aston-Jones G. & Cohen J. D. An integrative theory of locus coeruleus-norepinephrine function: adaptive gain and optimal performance. Annu Rev Neurosci 28, 403–450 (2005). 16022602 10.1146/annurev.neuro.28.061604.135709

[b7] Yu A. J. & Dayan P. Uncertainty, neuromodulation, and attention. Neuron 46, 681–692 (2005). 15944135 10.1016/j.neuron.2005.04.026

[b8] Mather M., Clewett D., Sakaki M. & Harley C. W. Norepinephrine ignites local hot spots of neuronal excitation: How arousal amplifies selectivity in perception and memory. Behav Brain Sci, 1–100, doi: 10.1017/S0140525X15000667 (2015). PMC5830137 26126507

[b9] Amemiya S. & Redish A. D. Manipulating Decisiveness in Decision Making: Effects of Clonidine on Hippocampal Search Strategies. J Neurosci 36, 814–827 (2016). 26791212 10.1523/JNEUROSCI.2595-15.2016 PMC4719017

[b10] Doya K. Metalearning and neuromodulation. Neural Netw 15, 495–506 (2002). 12371507 10.1016/s0893-6080(02)00044-8

[b11] Uhlen S., Muceniece R., Rangel N., Tiger G. & Wikberg J. E. Comparison of the binding activities of some drugs on alpha 2A, alpha 2B and alpha 2C-adrenoceptors and non-adrenergic imidazoline sites in the guinea pig. Pharmacology & toxicology 76, 353–364 (1995). 7479575 10.1111/j.1600-0773.1995.tb00161.x

[b12] Mao Z. M., Arnsten A. F. & Li B. M. Local infusion of an alpha-1 adrenergic agonist into the prefrontal cortex impairs spatial working memory performance in monkeys. Biological psychiatry 46, 1259–1265 (1999). 10560031 10.1016/s0006-3223(99)00139-0

[b13] Arnsten A. F. & Goldman-Rakic P. S. Analysis of alpha-2 adrenergic agonist effects on the delayed nonmatch-to-sample performance of aged rhesus monkeys. Neurobiol Aging 11, 583–590 (1990). 1980719 10.1016/0197-4580(90)90021-q

[b14] Franowicz J. S. & Arnsten A. F. The alpha-2a noradrenergic agonist, guanfacine, improves delayed response performance in young adult rhesus monkeys. Psychopharmacology 136, 8–14 (1998). 9537677 10.1007/s002130050533

[b15] Caetano M. S. . Noradrenergic control of error perseveration in medial prefrontal cortex. Frontiers in Integrative Neuroscience 6, 125 (2012). 23293590 10.3389/fnint.2012.00125 PMC3534184

[b16] Kim S., Bobeica I., Gamo N. J., Arnsten A. F. & Lee D. Effects of alpha-2A adrenergic receptor agonist on time and risk preference in primates. Psychopharmacology 219, 363–375 (2012). 21979441 10.1007/s00213-011-2520-0 PMC3269972

[b17] Seu E., Lang A., Rivera R. J. & Jentsch J. D. Inhibition of the norepinephrine transporter improves behavioral flexibility in rats and monkeys. Psychopharmacology 202, 505–519 (2009). 18604598 10.1007/s00213-008-1250-4 PMC2634830

[b18] Kawaura K., Karasawa J., Chaki S. & Hikichi H. Stimulation of postsynapse adrenergic alpha2A receptor improves attention/cognition performance in an animal model of attention deficit hyperactivity disorder. Behav Brain Res 270, 349–356 (2014). 24882610 10.1016/j.bbr.2014.05.044

[b19] Aoki C., Go C. G., Venkatesan C. & Kurose H. Perikaryal and synaptic localization of alpha 2A-adrenergic receptor-like immunoreactivity. Brain Res 650, 181–204 (1994). 7953684 10.1016/0006-8993(94)91782-5

[b20] Barth A. M., Vizi E. S., Zelles T. & Lendvai B. Alpha2-adrenergic receptors modify dendritic spike generation via HCN channels in the prefrontal cortex. J Neurophysiol 99, 394–401 (2008). 18003878 10.1152/jn.00943.2007

[b21] Ji X. H., Ji J. Z., Zhang H. & Li B. M. Stimulation of alpha2-adrenoceptors suppresses excitatory synaptic transmission in the medial prefrontal cortex of rat. Neuropsychopharmacology 33, 2263–2271 (2008). 17957212 10.1038/sj.npp.1301603

[b22] Yi F., Liu S. S., Luo F., Zhang X. H. & Li B. M. Signaling mechanism underlying alpha2A -adrenergic suppression of excitatory synaptic transmission in the medial prefrontal cortex of rats. Eur J Neurosci 38, 2364–2373 (2013). 23701442 10.1111/ejn.12257

[b23] Engberg G. & Eriksson E. Effects of alpha 2-adrenoceptor agonists on locus coeruleus firing rate and brain noradrenaline turnover in N-ethoxycarbonyl-2-ethoxy-1,2-dihydroquinoline (EEDQ)-treated rats. Naunyn-Schmiedeberg’s archives of pharmacology 343, 472–477 (1991). 10.1007/BF00169548 1652697

[b24] Jakala P. . Guanfacine, but not clonidine, improves planning and working memory performance in humans. Neuropsychopharmacology 20, 460–470 (1999). 10192826 10.1016/S0893-133X(98)00127-4

[b25] Jakala P. . Guanfacine and clonidine, alpha 2-agonists, improve paired associates learning, but not delayed matching to sample, in humans. Neuropsychopharmacology 20, 119–130 (1999). 9885792 10.1016/S0893-133X(98)00055-4

[b26] Muller U. . Lack of effects of guanfacine on executive and memory functions in healthy male volunteers. Psychopharmacology 182, 205–213 (2005). 16078088 10.1007/s00213-005-0078-4

[b27] Scahill L. . A placebo-controlled study of guanfacine in the treatment of children with tic disorders and attention deficit hyperactivity disorder. The American journal of psychiatry 158, 1067–1074 (2001). 11431228 10.1176/appi.ajp.158.7.1067

[b28] Huys Q. J. M., Maia T. V. & Frank M. J. Computational psychiatry as a bridge from neuroscience to clinical applications. Nat Neurosci 19, 404–413 (2016). 26906507 10.1038/nn.4238 PMC5443409

[b29] Stephan K. E. . Computational neuroimaging strategies for single patient predictions. NeuroImage in press (2015). 10.1016/j.neuroimage.2016.06.038 27346545

[b30] Arnsten A. F., Cai J. X. & Goldman-Rakic P. S. The alpha-2 adrenergic agonist guanfacine improves memory in aged monkeys without sedative or hypotensive side effects: evidence for alpha-2 receptor subtypes. J Neurosci 8, 4287–4298 (1988). 2903226 10.1523/JNEUROSCI.08-11-04287.1988 PMC6569464

[b31] Callado L. F. & Stamford J. A. Alpha2A- but not alpha2B/C-adrenoceptors modulate noradrenaline release in rat locus coeruleus: voltammetric data. Eur J Pharmacol 366, 35–39 (1999). 10064149 10.1016/s0014-2999(98)00889-9

[b32] Millan M. J. . Cognitive dysfunction in psychiatric disorders: characteristics, causes and the quest for improved therapy. Nature reviews. Drug discovery 11, 141–168 (2012). 22293568 10.1038/nrd3628

[b33] Niv Y. . Reinforcement learning in multidimensional environments relies on attention mechanisms. J Neurosci 35, 8145–8157 (2015). 26019331 10.1523/JNEUROSCI.2978-14.2015 PMC4444538

[b34] Balcarras M., Ardid S., Kaping D., Everling S. & Womelsdorf T. Attentional Selection Can Be Predicted by Reinforcement Learning of Task-relevant Stimulus Features Weighted by Value-independent Stickiness. J Cogn Neurosci 28, 333–349 (2016). 26488586 10.1162/jocn_a_00894

[b35] Redish A. D., Jensen S., Johnson A. & Kurth-Nelson Z. Reconciling reinforcement learning models with behavioral extinction and renewal: implications for addiction, relapse, and problem gambling. Psychol Rev 114, 784–805 (2007). 17638506 10.1037/0033-295X.114.3.784

[b36] Nassar M. R. . Rational regulation of learning dynamics by pupil-linked arousal systems. Nat Neurosci 15, 1040–1046 (2012). 22660479 10.1038/nn.3130 PMC3386464

[b37] O’Reilly J. X. . Dissociable effects of surprise and model update in parietal and anterior cingulate cortex. Proc Natl Acad Sci USA 110, 3660–3669 (2013). 10.1073/pnas.1305373110 PMC3780876 23986499

[b38] Shenhav A., Botvinick M. M. & Cohen J. D. The expected value of control: an integrative theory of anterior cingulate cortex function. Neuron 79, 217–240 (2013). 23889930 10.1016/j.neuron.2013.07.007 PMC3767969

[b39] Womelsdorf T. & Everling S. Long-Range Attention Networks: Circuit Motifs Underlying Endogenously Controlled Stimulus Selection. Trends Neurosci 38, 682–700 (2015). 26549883 10.1016/j.tins.2015.08.009

[b40] Yang Y. . Nicotinic alpha7 receptors enhance NMDA cognitive circuits in dorsolateral prefrontal cortex. Proc Natl Acad Sci USA 110, 12078–12083 (2013). 23818597 10.1073/pnas.1307849110 PMC3718126

[b41] Aston-Jones G., Rajkowski J. & Cohen J. Role of locus coeruleus in attention and behavioral flexibility. Biological psychiatry 46, 1309–1320 (1999). 10560036 10.1016/s0006-3223(99)00140-7

[b42] Cole B. J. & Robbins T. W. Forebrain norepinephrine: role in controlled information processing in the rat. Neuropsychopharmacology 7, 129–142 (1992). 1418302

[b43] Dalley J. W., Cardinal R. N. & Robbins T. W. Prefrontal executive and cognitive functions in rodents: neural and neurochemical substrates. Neuroscience and biobehavioral reviews 28, 771–784 (2004). 15555683 10.1016/j.neubiorev.2004.09.006

[b44] Devauges V. & Sara S. J. Activation of the noradrenergic system facilitates an attentional shift in the rat. Behav Brain Res 39, 19–28 (1990). 2167690 10.1016/0166-4328(90)90118-x

[b45] Connor D. F., Arnsten A. F., Pearson G. S. & Greco G. F. Guanfacine extended release for the treatment of attention-deficit/hyperactivity disorder in children and adolescents. Expert opinion on pharmacotherapy 15, 1601–1610 (2014). 24992513 10.1517/14656566.2014.930437

[b46] Sallee F. R. . Guanfacine extended release in children and adolescents with attention-deficit/hyperactivity disorder: a placebo-controlled trial. J Am Acad Child Adolesc Psychiatry 48, 155–165 (2009). 19106767 10.1097/CHI.0b013e318191769e

[b47] Steere J. C. & Arnsten A. F. The alpha-2A noradrenergic receptor agonist guanfacine improves visual object discrimination reversal performance in aged rhesus monkeys. Behav Neurosci 111, 883–891 (1997). 9383511 10.1037//0735-7044.111.5.883

[b48] Doya K. Modulators of decision making. Nat Neurosci 11, 410–416 (2008). 18368048 10.1038/nn2077

[b49] Wang X. J. & Krystal J. H. Computational psychiatry. Neuron 84, 638–654 (2014). 25442941 10.1016/j.neuron.2014.10.018 PMC4255477

[b50] Wiecki T. V. . A Computational Cognitive Biomarker for Early-Stage Huntington’s Disease. PLoS One 11, e0148409, doi: 10.1371/journal.pone.0148409 (2016). 26872129 PMC4752511

[b51] Huys Q. J., Pizzagalli D. A., Bogdan R. & Dayan P. Mapping anhedonia onto reinforcement learning: a behavioural meta-analysis. Biol Mood Anxiety Disord 3, 12 (2013). 23782813 10.1186/2045-5380-3-12 PMC3701611

[b52] Gershman S. J. & Niv Y. Learning latent structure: carving nature at its joints. Curr Opin Neurobiol 20, 251–256 (2010). 20227271 10.1016/j.conb.2010.02.008 PMC2862793

[b53] Voon V. . Disorders of compulsivity: a common bias towards learning habits. Mol Psychiatry 20, 345–352 (2015). 24840709 10.1038/mp.2014.44 PMC4351889

[b54] Maia T. V. & Frank M. J. From reinforcement learning models to psychiatric and neurological disorders. Nature Neuroscience 14, 154–162 (2011). 21270784 10.1038/nn.2723 PMC4408000

[b55] Adams R. A., Huys Q. J. M. & Roiser J. P. Computational Psychiatry: towards a mathematically informed understanding of mental illness. Journal of Neurology, Neurosurgery, and Psychiatry 87, 53–63 (2015). 10.1136/jnnp-2015-310737 PMC4717449 26157034

[b56] Schlagenhauf F. . Striatal dysfunction during reversal learning in unmedicated schizophrenia patients. NeuroImage 89, 171–180 (2014). 24291614 10.1016/j.neuroimage.2013.11.034 PMC3991847

[b57] Harlé K. M. . Bayesian neural adjustment of inhibitory control predicts emergence of problem stimulant use. Brain 138, 3413–3426 (2015). 26336910 10.1093/brain/awv246 PMC4731415

[b58] Zhang J. . Different decision deficits impair response inhibition in progressive supranuclear palsy and Parkinson’s disease. Brain 139, 161–173 (2016). 26582559 10.1093/brain/awv331 PMC4949391

[b59] Frank M. J. . fMRI and EEG Predictors of Dynamic Decision Parameters during Human Reinforcement Learning. Journal of Neuroscience 35, 485–494 (2015). 25589744 10.1523/JNEUROSCI.2036-14.2015 PMC4293405

[b60] Smith A. C. & Brown E. N. Estimating a state-space model from point process observations. Neural Comput 15, 965–991 (2003). 12803953 10.1162/089976603765202622

[b61] Wilson R. C. & Niv Y. Inferring relevance in a changing world. Frontiers in human neuroscience 5, 189 (2011). 22291631 10.3389/fnhum.2011.00189 PMC3264902

[b62] Rämä P. . Medetomidine, atipamezole, and guanfacine in delayed response performance of aged monkeys. Pharmacology Biochemistry and Behavior 55, 415–422 (1996). 10.1016/s0091-3057(96)00111-6 8951983

[b63] Arnsten A. F. T. & Contant T. A. Alpha-2 adrenergic agonists decrease distractibility in aged monkeys performing the delayed response task. Psychopharmacology 108, 159–169 (1992). 1357704 10.1007/BF02245302

[b64] O’Neill J. . Effects of guanfacine on three forms of distraction in the aging macaque. Life Sciences 67, 877–885 (2000). 10946847 10.1016/s0024-3205(00)00681-0

[b65] Wang M., Ji J.-Z. & Li B.-M. The α2A-Adrenergic Agonist Guanfacine Improves Visuomotor Associative Learning in Monkeys. Neuropsychopharmacology 29, 86–92 (2004). 12931141 10.1038/sj.npp.1300278

[b66] Witte E. a. & Marrocco R. T. Alteration of brain noradrenergic activity in rhesus monkeys affects the alerting component of covert orienting. Psychopharmacology 132, 315–323 (1997). 9298508 10.1007/s002130050351

[b67] Decamp E., Clark K. & Schneider J. S. Effects of the alpha-2 adrenoceptor agonist guanfacine on attention and working memory in aged non-human primates. European Journal of Neuroscience 34, 1018–1022 (2011). 21883531 10.1111/j.1460-9568.2011.07815.x PMC3177964

