# Supplementary material for: A computational psychiatry approach identifies how alpha-2A noradrenergic agonist Guanfacine affects feature-based reinforcement learning in the macaque
Source: Sci Rep. 2017 Jan 16;7:40606. doi: 10.1038/srep40606 (PMC5238510; doi:10.1038/srep40606)
Supplement: Supplementary Information [file srep40606-s1.pdf]

Supplementary Information for

A computational psychiatry approach identifies how alpha-2A noradrenergic agonist Guanfacine affects feature-based reinforcement learning in the macaque

Authors: S.A. Hassani[1*], M. Oemisch[1*], M. Balcarras[1], S. Westendorff[1], S. Ardid[2], M.A. van der Meer[3], P. Tiesinga[4] and T. Womelsdorf[1]

Authors Institutions: *[1] Department of Biology, Centre for Vision Research, York University, Toronto, Ontario M6J 1P3, Canada; [2] Department of Mathematics, Boston University, Boston, MA 02215, USA; [3]Department of Psychological and Brain Sciences, Dartmouth College, Hanover, NH 03755,USA. [4]Department of Neuroinformatics, Donders Centre for Neuroscience, Radboud University Nijmegen, 6525 AJ, Nijmegen, The Netherlands*

[*] *These authors contributed equally to this work.*

Supplementary Results 1: Early error commissions, attentional lapses and perseverations were unaffected by Guanfacine.

Premature fixation breaks are errors committed during covert attention deployment to one stimulus (i.e. after color onset) and before the change event (the dimming). During the 19 week testing period we found that on average 8.10 % (SE: 0.98) and 13.05 % (SE: 1.35) of trials were premature fixation breaks in the Control and Guanfacine sessions respectively, which is significantly different (Wilcoxon rank sum test, $p < 0.01$), similar to what we had found for higher doses in the dose identification phase of our experiment (see above). We next asked whether erroneous saccadic responses during the time of the stimulus change varied between

drug conditions. The task design included trials in which the rewarded stimulus dimmed before, at the same time, and after the unrewarded stimulus. The pilot dose identification testing suggested that Guanfacine reduces these errors which would be a strong indication that Guanfacine acts by reducing interference from salient but irrelevant stimulus events as changes of the unrewarded stimulus when it changed before, or at the same time as the rewarded stimulus. However, we found that outside the pilot dose testing phase, the proportion of errors committed in Control and Guanfacine conditions was on average not different between those trials in which dimming occurred (1) in the unrewarded stimulus before the rewarded stimulus (Control: 0.26 (SE: 0.01); Guanfacine: 0.24 (SE: 0.01)), (2) at the same time in rewarded and unrewarded stimuli (Control: 0.30 (SE: 0.01); Guanfacine: 0.30 (SE: 0.01)), or (3) in the rewarded stimulus before the unrewarded stimulus (Control: 0.17 (SE 0.02); Guanfacine: 0.16 (SE 0.02)). To ensure that we did not miss an effect that occurred only in subsets of trials, we performed this error-saccade analysis also within sliding windows of 5 trials from trial 1 to 30 since the reversal, but did not find differences between Control and Guanfacine conditions for any trial window (data not shown).

We next confirmed the negative results from the error type analysis by comparing the performance accuracy (rather than the proportion of error subtype) in trials when rewarded and non-rewarded dimming occurred at the same time and found that Control and Guanfacine days were in fact not different (overall accuracy on same-time dimming trials: Control: 64.89% (SE: 1.97); Guanfacine: 62.32% (SE: 1.44), Wilcoxon rank sum test, $p = 0.5019$). Likewise, there was no difference in accuracy on trials when the unrewarded stimulus dimmed before the rewarded stimulus (Control: 71.06% (SE: 1.76); Guanfacine: 69.62% (SE: 2.32), Wilcoxon rank sum test, $p = 0.9302$), and neither when the rewarded stimulus dimmed before the unrewarded stimulus

(Control: 60.46% (SE: 01.84); Guanfacine: 58.68% (SE: 01.49), Wilcoxon rank sum test, $p = 0.4137$). We analyzed accuracy also with a sliding window of 5 trials from trial 1 to 30 since the reversal and did not found apparent differences between Control and Guanfacine conditions for any trial window (data not shown).

The 19 week testing period provided sufficient data to test for variations of rare behavioral errors such as perseveration errors. The monkey showed perseveration of unrewarded choices following an unrewarded trial resulting from the wrong color choice in 12.6-14.4% of all unrewarded choices (sequences of successive error trials such as CEE, CEEE, …, CEE_n). These color-based perseveration errors did not differ between Control (12.6%, SE 3.1) and Guanfacine (14.4%, SE 2.9) days (Wilcoxon rank sum test, $p = 0.066$). To test whether the animal persevered on features other than color we calculated the same percent perseverations (successively unrewarded choices) on the motion direction (e.g. successive unrewarded downward saccades to the dimming) and the stimulus location (e.g. successive unrewarded choice on the motion direction of the stimulus in the right visual field), and on combinations of all features (e.g. successive erroneous choice on the stimulus with the same color on the right side moving downward). There was no significant difference in the percentage of perseveration on motion direction (Control: 9.38% (SE: 3.10); Guanfacine: 10.75% (SE: 2.20), Wilcoxon rank sum test, $p = 0.0798$), stimulus location (Control: 10.56% (SE: 03.08); Guanfacine: 11.78% (SE 3.09); Wilcoxon rank sum test, $p = 0.2201$), or conjunctions of stimulus features between Control and Guanfacine sessions (all tests for differences, $p > 0.05$).

Supplementary Results 2: Consistency of learning benefit with Guanfacine across blocks in the experimental session.

This result of enhanced learning success during the actual learning period of the task could be robust across all blocks of a session on Guanfacine days. In another scenario, it could emerge particularly at later stages of a training session where sustained attention and motivation may benefit most from enhanced noradrenergic action. Alternatively, it may be evident only during early blocks in which the brain concentration of Guanfacine action will be relatively higher than late in the session (**Supplementary Fig. 2A,** for pharmacokinetic results of Guanfacine, see **Supplementary Results 3**). We tested these alternatives by calculating the average learning trials for sets of 4 adjacent blocks relative to the first block of the day with a sliding window until block eight (which is the average number of performed blocks, see above). We then took the reverse approach and calculated the average learning trials for blocks relative to the last block of the day (see Methods). This procedure ensured that a maximal number of blocks contributed to the estimated learning success across the day. We found that relative to the first block of the day, seven of eight block sets showed an average faster learning on Guanfacine days than on Control days (**Supplementary Fig. 2B**). In contrast, we found that only four of seven block sets since the last block of a day's session showed faster average learning in Guanfacine than Control sessions. To test whether the learning effect is still robust across the behavioral sessions we used permutation statistics) (see **Supplementary Methods**), finding that the likelihood to observe faster learning in Guanfacine versus Control block sets in 11 of 15 possible block sets is significantly larger than chance (permutation statistics, $p < 0.001$) (**Supplementary Fig. 2B**).

Supplementary Results 3: Characterization of Guanfacine's pharmacokinetics using High Performance Liquid Chromatography (HPLC) and Mass Spectrometry (MS).

We characterized the pharmacokinetics of Guanfacine at the dose that we identified to be the behaviourally efficient dose (0.75mg/kg) in the dose testing phase of the experiment. In order to quantify Guanfacine's metabolism and degradation rate within the macaque model we devised a protocol in which blood samples were taken every 40 minutes for 4 hours after Guanfacine injection and the blood concentration of Guanfacine was measured using High Performance Liquid Chromatography (HPLC) and Mass Spectrometry (MS) similar to previous studies in humans[1]. Using this method, we acquired a resolution capable of detecting as little as 30 femtomoles of drug. The procedure started with the placement of a catheder for later blood sample extraction using light anaesthesia (Dexdomitor and Ketamine) reversed with Antisedan. The awake animal was then seated in a custom primate chair and engaged in watching a movie while 300 µl blood samples were taken every 40 minutes for 4 hours (0, 40, 80, 120, 160, 200, and 240min). This time frame was well within the range of all recording sessions relative to injection. The blood samples were left in room temperature until clotting was observed, typically 30-60min, and then transported to a 4 °C fridge. Upon the final sample extraction, all blood samples were transported to a centrifuge where they were spun at 2000 rpm for 40 minutes in order to separate the serum. The serum was then aliquoted and spin filtered (3kDa molecular weight cut off) and had acid added to the sample to help with preservation. The samples were then frozen at -80 °C until the HPLC protocol was applied. Each sample provided triplicate results (technical replicate) and was loaded into the HPLC into a c18 reversed phase column where unbound protein and molecules were washed out for 15 min with 5% aceto-nitrile. Then a quick ramp up to 80% aceto-nitrile (20 min process with a period of 5 min with 80% aceto-nitrile) released the bound compounds in the HPLC column. Then the washed solution was subjected to a multi reaction monitoring protocol using a MS causing the breaking of Guanfacine

into two component fragments (control experiments with drug only samples were already done in order to quantify MS peaks expected by Guanfacine) that were used to identify and quantify Guanfacine blood concentrations.

The results of this protocol yielded an expected half life of Guanfacine of 43.23 min with a plateau of Guanfacine concentrations 2 hours after injection at <10µg/kg which is when most of the experimental data collection started (across sessions the average time was 150.8 minutes (SE: 0.88). To our knowledge, Guanfacine concentrations have only been described in humans[1,2]. And using the orally administered extended release versions with half lives of ~17 hours based off of plasma concentrations. Most macaque papers cite Arnsten et al., (1988)[3] in order to justify their concentration use. Almost all papers with systemic administration do so at a 2h benchmark prior to testing.

Supplementary Results 4: Comparing early and late control session performance to discern possible longer-term effects of drug administration

To test whether there were overall changes in behavioral performance over the 19 weeks drug and control period testing, we analyzed early and late control session performance separately. If similar control session performance remained for early and late periods, it would suggest that administering the drug over longer periods does not have adverse effects on overall performance. We found that early (first nine sessions) and late (last 10 sessions) control sessions did not differ with regard to the number of performed reversal blocks (Early / late: 8.1 SE: 0.46 / 7.8 SE: 0.61), the number of blocks with learning within 30 trials (Early / late: 5.3 SE: 0.69 / 5.0 SE: 0.47), the median trial at which the ideal observer procedure detected learning (Early / late: 11 SE: 1.1 / 12 SE: 0.96) (all n.s.). Likewise, early and late sessions did not show differences of the probability

of rewarded choices (quantified using the Smith algorithm used for Fig. 3 of the main text) as a function of the trial number since reversal (randomization test with multiple comparison correction for the number of trials).

Supplementary Methods

Behavioural analysis of learning trials

Analysis was performed with custom MATLAB code (Mathworks, Natick, MA), utilizing functionality from the open-source fieldtrip toolbox (http://www.ru.nl/fcdonders/fieldtrip/). To identify at which trial during a block the monkey showed statistically reliable learning we analyzed the monkeys' trial-by-trial choice dynamics using the state–space framework introduced by Smith and Brown[7], and implemented by Smith et al.[8]. This framework entails a state equation that describes the internal learning process as a hidden Markov or latent process and is updated with each trial. The learning state process estimates the probability of a correct (rewarded) choice in each trial and thus provides the learning curve of subjects (see e.g.[9]). The algorithm estimates learning from the perspective of an ideal observer that takes into account all trial outcomes of subjects' choices in a block of trials to estimate the probability that the outcome in a single trial is correct or incorrect. The ideal observer perspective corresponds to smoothing in the Kalman filter context[10]. This probability is then used to calculate the confidence range of observing a correct response. We defined the learning trial as the earliest trial in a block at which the lower confidence bound of the probability for a correct response exceeded the $p = 0.5$ chance level.

More specifically, the algorithm defines the learning state process as a random walk whereby each trial's probability of a correct response depends on the previous trials probability, or on the chance level in case there was no previous trial's probability i.e. at the beginning of blocks. According to this formulation, the subject's choices across trials follow a random strategy. The mean of the random process reflects the current probability for a correct response. The variance of the random process determines how fast the learning state process can change from trial to trial and thus, how rapidly learning can take place (see[8]). The Expectation-Maximization (EM) algorithm is used to estimate the mean and variance of the random process by maximum likelihood estimation[11] to derive the probability to observe a correct response in each trial as a function of the trial number[7]. A forward filter estimates the variance and mean of the value of the Gaussian Random Variable from the first trial to the current trial. This forward process reflects a state estimate from the perspective of the subject performing the task. An additional smoothing algorithm takes the perspective of an ideal observer and estimates the current trials mean and variance of the state process using data from all trials. The estimates of both, the forward filter and the smoothing process are then used to calculate the probability density for the correct response probability at each trial. Please see Smith et al.[8] equations 2.1 to 2.4 for details. The aforementioned procedure provides the learning curve, i.e. it provides for each trial the probability of a correct response given the sequence of correct and incorrect choices of the monkey. To identify the first trial in a block at which an ideal observer knows with $p \geq 0.95$ confidence that learning has taken place, we calculated the lower confidence bound and identified the first trial where it exceeded the $p=0.5$ chance level, the first 'IO95' learning trial (see[8]). This corresponds to a 0.95 confidence level for an ideal observer to identify learning.

Supplementary Figures

Supplementary Fig 1. Examples of learning at varying speeds estimated with an ideal observer estimate of choice confidence. (**A**) Four example blocks with learning within the first eleven trials, denoted as 'fast learning'. Upper panel show the reward probability (solid red line) estimated with an expectation maximization algorithm and the 95% confidence levels (dashed red line). The learning trial is defined as the first trial at which the lower bound 95% confidence level exceeds and never dips below the 0.5 chance probability with which an ideal observer can estimate that consistent learning has occurred. Squares on top of the panel highlight whether the choice was rewarded (black) or unrewarded (grey). Bottom panel shows for the same block the proportion of rewarded choices calculated with a running average sliding window of up to eight trials in the past. The text denotes overall percent correct (rewarded) performance across all 35 trials. (**B**) Same format as (*A*) but for blocks with 'slow learning', defined by learning trials between 12 and 35 within a block. (**C**) Same format as (*A,B*) but for blocks with no learning of the new color-reward association evident statistically in the first 35 blocks.

Supplementary Fig 2. Consistency of reversal learning benefit on Guanfacine days versus Control days. (**A**) Illustration of the time of drug/control injection and the time range with behavioural performance. (**B**) Average learning trials (*y-axis*) for reversal learning blocks on Control days (*black*) and Guanfacine days (*red*) relative to the first block performed in the day (*left panel*), and relative to the last performed block in the day (*right panel*). A total of 11 of 15 blocks show on average an earlier learning trial in blocks on Guanfacine days versus Control days, which is a statistically significant difference (randomization test, $p = 0.024$).

Supplementary Fig 3. Performance for the seven RL models with worse log likelihood and inferior sum of squared error for the most-predictive RL model (**A,B**) Proportion of rewarded choices for the monkey and model across trials since reversal in Guanfacine (*A*) and control (*B*) sessions. The model simulations are based on the best model, the *Feature-Weighting + Decay RL model* (*see* Fig. 4 and methods). The inset shows the sum of squared errors (SSD) between the proportion of correct monkey choices (*x-axis*) and the choice probability of the model across trials since reversal. Data on the diagonal in these plots would indicate a perfect match between model choices and subject choices. (**C**) The average parameter values for n=100 models fitted to subsets of 80% (cross-validation) reversal blocks for the Guanfacine (*red*) and control (*blue*) sessions. Error bars denote STD. Three stars denote significance at $p < 0.001$ after Bonferroni correction). See Figure 4 in the main text for a comparison of optimization scores across models.

Supplementary References

1 Boellner, S. W., Pennick, M., Fiske, K., Lyne, A. & Shojaei, A. Pharmacokinetics of a guanfacine extended-release formulation in children and adolescents with attention-deficit-hyperactivity disorder. *Pharmacotherapy* **27**, 1253-1262 (2007).

2 Kiechel, J. R. Pharmacokinetics and metabolism of guanfacine in man: a review. *Br J Clin Pharmacol* **10 Suppl 1**, 25S-32S (1980).

3 Arnsten, A. F., Cai, J. X. & Goldman-Rakic, P. S. The alpha-2 adrenergic agonist guanfacine improves memory in aged monkeys without sedative or hypotensive side effects: evidence for alpha-2 receptor subtypes. *J Neurosci* **8**, 4287-4298 (1988).

4 Steere, J. C. & Arnsten, A. F. The alpha-2A noradrenergic receptor agonist guanfacine improves visual object discrimination reversal performance in aged rhesus monkeys. Behav Neurosci **111**, 883-891 (1997).

5 Franowicz, J. S. & Arnsten, A. F. The alpha-2a noradrenergic agonist, guanfacine, improves delayed response performance in young adult rhesus monkeys. Psychopharmacology **136**, 8-14 (1998).

6 O'Neill, J., Fitten, L. J., Siembieda, D. W., Ortiz, F. & Halgren, E. Effects of guanfacine on three forms of distraction in the aging macaque. Life Sci **67**, 877-885 (2000).

7 Smith, A. C. & Brown, E. N. Estimating a state-space model from point process observations. Neural Comput **15**, 965-991 (2003).

8 Smith, A. C. et al. Dynamic analysis of learning in behavioral experiments. J Neurosci **24**, 447-461 (2004).

9 Balcarras, M., Ardid, S., Kaping, D., Everling, S. & Womelsdorf, T. Attentional Selection Can Be Predicted by Reinforcement Learning of Task-relevant Stimulus Features Weighted by Value-independent Stickiness. J Cogn Neurosci **28**, 333-349 (2016).

10 Rauch, H. E., Tung, F. & Striebel, C. T. Maximum likelihood estimates of linear dynamic systems. AIAA **3**, 1445–1450 (1965).

11 Dempster, A. P., Laird, N. M. & Rubin, D. B. Maximum Likelihood from Incomplete Data Via Em Algorithm. J Roy Stat Soc B Met **39**, 1-38 (1977).

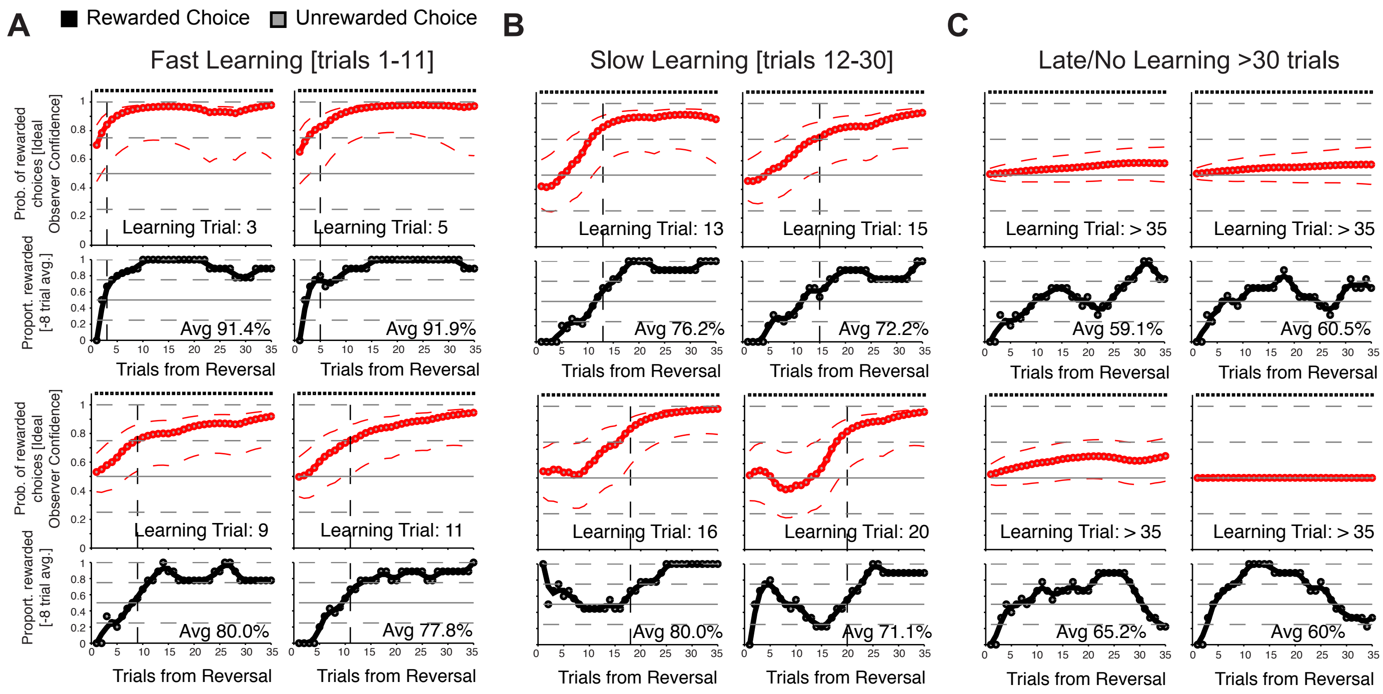

Supplementary Fig 1. Examples of learning at varying speeds estimated with an ideal observer estimate of choice confidence. (**A**) Four example blocks with learning within the first eleven trials, denoted as 'fast learning'. Upper panel show the reward probability (solid red line) estimated with an expectation maximization algorithm and the 95% confidence levels (dashed red line). The learning trial is defined as the first trial at which the lower bound 95% confidence level exceeds and never dips below the 0.5 chance probability with which an ideal observer can estimate that consistent learning has occurred. Squares on top of the panel highlight whether the choice was rewarded (black) or unrewarded (grey). Bottom panel shows for the same block the proportion of rewarded choices calculated with a running average sliding window of up to eight trials in the past. The text denotes overall percent correct (rewarded) performance across all 35 trials. (**B**) Same format as (*A*) but for blocks with 'slow learning', defined by learning trials between 12 and 35 within a block. (**C**) Same format as (*A*,*B*) but for blocks with no learning of the new color-reward association evident statistically in the first 35 blocks.

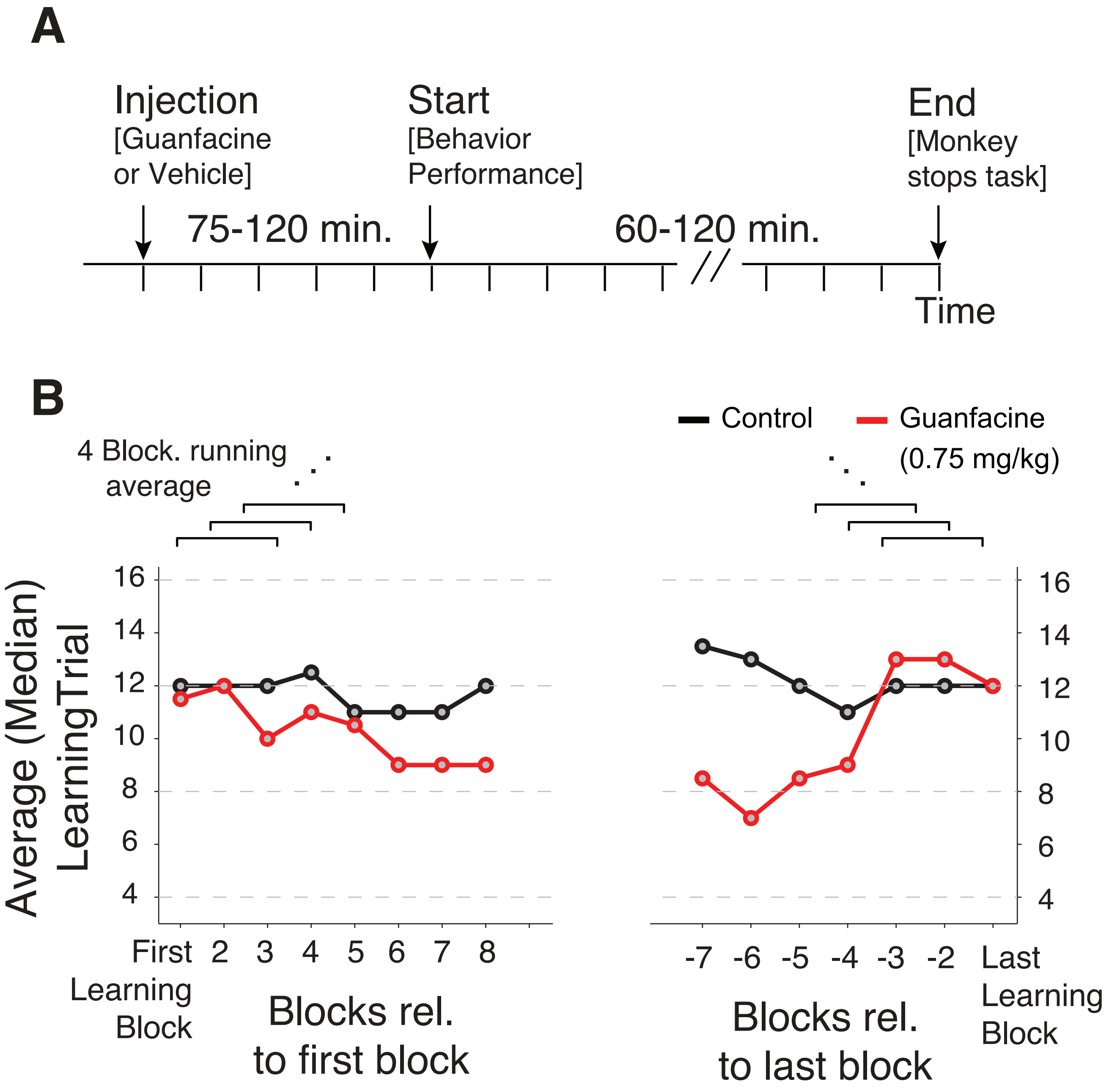

Supplementary Fig 2. Consistency of reversal learning benefit on Guanfacine days versus Control days. (**A**) Illustration of the time of drug/control injection and the time range with behavioural performance. (**B**) Average learning trials (y-axis) for reversal learning blocks on Control days (black) and Guanfacine days (red) relative to the first block performed in the day (left panel), and relative to the last performed block in the day (right panel). A total of 11 of 15 blocks show on average an earlier learning trial in blocks on Guanfacine days versus Control days, which is a statistically significant difference (randomization test, p = 0.024).

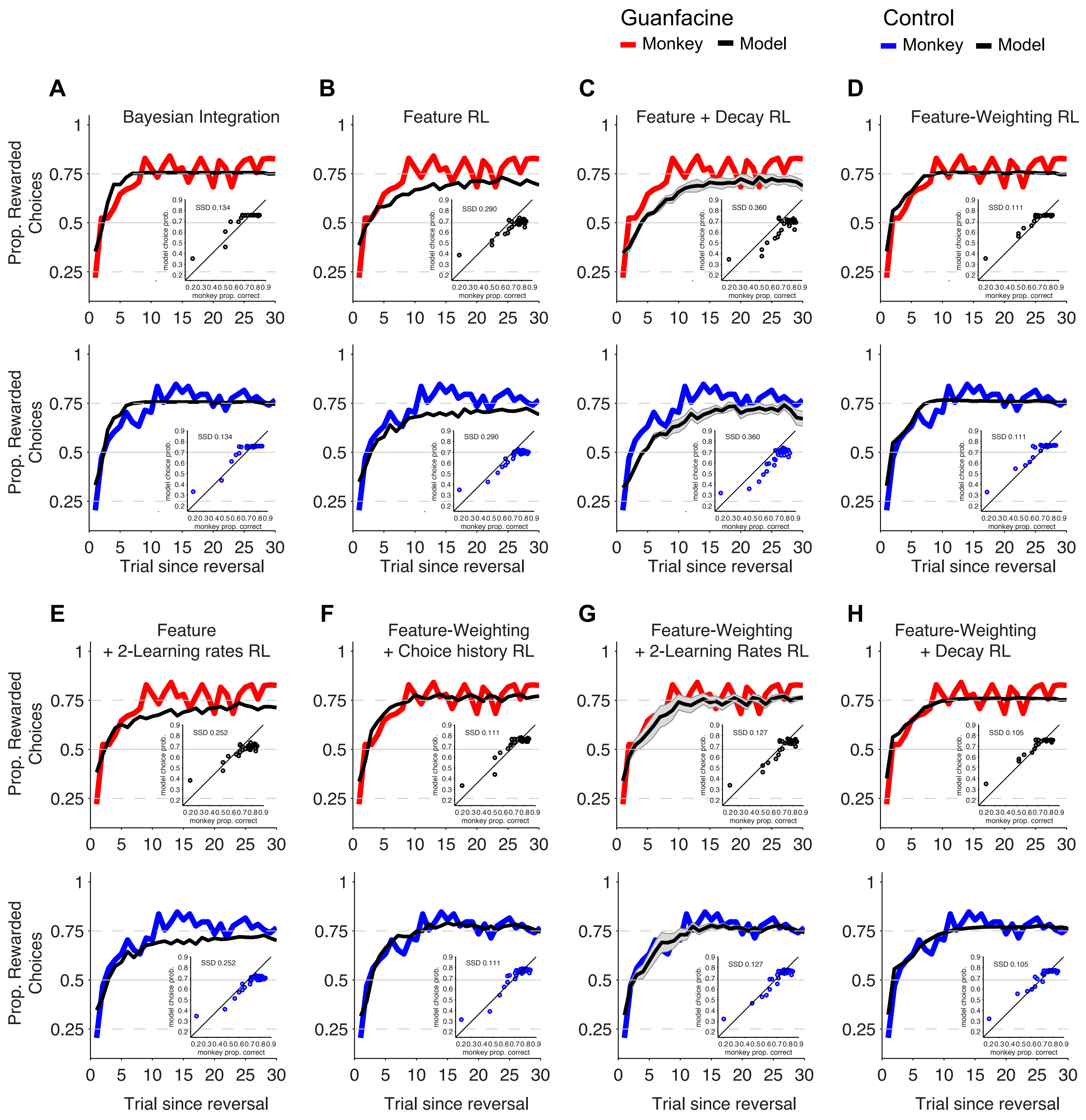

Supplementary Fig 3. Performance for the seven RL models with worse log likelihood and inferior sum of squared error for the most-predictive RL model (**A**,**B**) Proportion of rewarded choices for the monkey and model across trials since reversal in Guanfacine (*A*) and control (*B*) sessions. The model simulations are based on the best model, the Feature-Weighting + Decay RL model (see Fig. 4 and methods). The inset shows the sum of squared errors (SSD) between the proportion of correct monkey choices (x-axis) and the choice probability of the model across trials since reversal. Data on the diagonal in these plots would indicate a perfect match between model choices and subject choices. (**C**) The average parameter values for n=100 models fitted to subsets of 80% (cross-validation) reversal blocks for the Guanfacine (red) and control (blue) sessions. Error bars denote STD. Three stars denote significance at $p < 0.001$ after Bonferroni correction). See Figure 4 in the main text for a comparison of optimization scores across models.
